# Stroma-Dominant Colorectal Cancer Harbors CAF-Rich Spatial Architecture and EMT-Associated Cancer Cell Plasticity Detectable After Dissemination

**DOI:** 10.3390/biom16091261

**Published:** 2026-08-31

**Authors:** Shuji Kitagawa, Naoki Mimura, Hironobu Kambara, Masaaki Hori, Erina Yamanishi, Ayako Ogo, Tatsushi Shiomi, Kazuhiko Yoshimatsu, Tomio Ueno, Shuya Yano

**Affiliations:** 1 Department of Digestive Surgery, Kawasaki Medical School, Kurashiki 701-0192, Japan; kitagawashuji@med.kawasaki-m.ac.jp (S.K.); kanbara-hironobu@med.kawasaki-m.ac.jp (H.K.); masaaki1002@med.kawasaki-m.ac.jp (M.H.); kyoshsu@med.kawasaki-m.ac.jp (K.Y.); tommy@med.kawasaki-m.ac.jp (T.U.); 2 Department of Gastroenterological Surgery, Graduate School of Medicine, Dentistry and Pharmaceutical Sciences, Okayama University, Okayama 700-8558, Japan; mimuking0531@gmail.com; 3 Department of Pathology, Kawasaki Medical School, Kurashiki 701-0192, Japan

**Keywords:** colorectal cancer, spatial transcriptomics, malignant ascites, epithelial–mesenchymal transition, cancer-associated fibroblast, retained epithelial features, H&E morphology, CMS4

## Abstract

Colorectal cancers (CRCs) include stroma-dominant tumors with desmoplasia and differentiated, gland-forming tumors with little stroma. We asked whether this difference reflects stromal abundance alone or also involves a distinct cancer cell state. HEST-1K sections were classified as M-type (stroma-dominant; five patient/tissue units) or D-type (differentiated and cancer cell-dominant; seven units). Prespecified EMT/pEMT, ECM/integrin, YAP/TAZ-TEAD, and DTP/persister gene sets were examined across spatial, bulk, and single-cell datasets and patient-derived malignant ascites cultures. M-type tumors contained broader CAF-rich compartments and higher activity of all four programs in EPCAM/KRT-high regions, including epithelial-dense tumor cores. Activity was greatest near CAF-rich areas. In GSE39582, EMT/pEMT, ECM/integrin, DTP/persister, and invasive epithelial programs were independently associated with recurrence. Ascites-derived cultures from stroma-dominant tumors showed higher ECM/integrin and survival programs. Independent institutional analyses supported the public data findings: focused real-time PCR showed higher MAPK/TGF-beta-related gene expression in M-type-derived than in D-type-derived ascites cells, and IHC showed stronger epithelial CD44v6 expression in M-type primary tumors and metastatic lymph node lesions. CAF co-culture increased VIM-promoter activity. These findings characterize stroma-dominant CRC as a spatially organized ecosystem in which a CAF-rich compartment is associated with stress-adapted cancer cell programs. Its poor prognosis may therefore involve not only stromal abundance but also a morphology-associated cancer cell state detectable after dissemination.

## 1. Introduction

Colorectal cancer (CRC) shows marked variation in its stromal architecture. Some tumors contain broad desmoplastic stroma, whereas others are dominated by differentiated, gland-forming cancer cells. This distinction is clinically relevant because stromal expansion and cancer-associated fibroblast (CAF) activity are associated with invasion, treatment resistance, recurrence, and poor outcome [1,2,3]. Recent single-cell, spatial, and functional studies have further shown that CAFs comprise distinct phenotypic populations and can remodel the extracellular matrix (ECM), alter epithelial behavior, and promote cancer cell invasion [4,5]. A central unresolved issue is therefore whether stroma-dominant CRC reflects stromal expansion alone or also contains a distinct state in the cancer cells.

Bulk transcriptomic classifications have captured part of this biology. CMS4 CRC is characterized by a mesenchymal, matrix-rich transcriptome and adverse clinical behavior [6,7]. However, much of the CMS4 signal comes from non-malignant stromal cells [2,3], and intratumoral heterogeneity can further complicate bulk molecular classification [8]. More recent single-cell studies have identified distinct epithelial tumor cell states within conventional CMS classes and have shown that epithelial, fibroblast, and immune cell states change together during colorectal tumor development [9,10]. Bulk RNA-seq alone therefore cannot determine whether mesenchymal transcripts reflect CAF abundance, cancer cell plasticity, or coordinated changes in both compartments.

Spatially resolved studies have begun to define how these compartments are organized. Integration of single-cell and spatial transcriptomics can localize cell states and candidate interactions while preserving tissue architecture [11]. In CRC, spatial profiling has identified multicellular neighborhoods, tumor–stroma boundaries, and CAF-associated compartments linked to distinct epithelial and immune states [12,13,14,15]. These studies establish that stromal biology is spatially organized, but it remains unclear whether EPCAM/KRT-high cancer cell regions in stroma-dominant tumors carry reproducible adaptive programs and whether those programs are concentrated near CAF-rich areas.

Cancer-associated epithelial-to-mesenchymal transition (EMT) is rarely a complete conversion from an epithelial to a mesenchymal identity. Cancer cells frequently retain epithelial markers while acquiring matrix interaction, motility, wound response, inflammatory, and survival programs, resulting in hybrid or partial EMT states [16,17,18,19,20,21,22,23,24,25]. Epithelial–mesenchymal plasticity encompasses multiple intermediate states whose stability and reversibility are shaped by transcriptional, epigenetic, and microenvironmental regulation [26]. Matrix engagement can also activate YAP/TAZ-dependent mechanotransduction [27,28], while stress-adapted and drug-tolerant persister states can support survival under therapeutic or environmental pressure [29,30,31,32]. CRC organoid–CAF co-culture studies have shown that fibroblast exposure can increase partial-EMT features, ECM remodeling, and phenotypic heterogeneity in epithelial cancer cells [33,34]. Together, these observations provide a biological basis for examining EMT/pEMT, ECM/integrin, YAP/TAZ-TEAD, and DTP/persister programs at the tumor–stroma interface.

Whether morphology-associated cancer cell features remain detectable after dissemination is less clear. Specific CRC cell states can contribute to metastatic recurrence and subsequently regenerate heterogeneous metastatic tumors [35]. Malignant ascites-derived cultures provide a complementary setting in which disseminated cells can be examined after expansion outside the architecture of the primary tumor. We therefore asked whether cultures derived from stroma-dominant and differentiated, cancer cell-dominant primary tumors showed different transcriptomic features under comparable ex vivo conditions.

To address these questions, we combined routine histology with HEST-1K spatial transcriptomics, public bulk and single-cell RNA-seq cohorts, patient-derived malignant ascites culture RNA-seq, focused real-time PCR, IHC, and CAF co-culture experiments. HEST-1K provides paired spatial transcriptomic profiles, H&E whole-slide images, and standardized metadata across multiple tissues and platforms [36]. Public bulk analyses included TCGA and GSE39582 [37,38], while the single-cell analyses included independent CRC atlases such as that reported by Lee et al. [39]. Prespecified EMT/pEMT, ECM/integrin, YAP/TAZ-TEAD, and DTP/persister gene sets were fixed before analysis. Spatial analyses separated CAF-rich compartments from EPCAM/KRT-high cancer cell regions, whereas the other datasets tested related programs across clinical cohorts, individual cell types, disseminated cultures, and experimental CAF exposure.

## 2. Materials and Methods

### 2.1. Bulk Cohorts and H&E Morphology

TCGA-COAD and TCGA-READ [37] formed the primary bulk cohort linking diagnostic H&E images, RNA-seq profiles, CMS labels, and clinical outcomes. Cases with available diagnostic whole-slide H&E images were reviewed at the case level using four prespecified morphological categories: differentiated/gland-forming (D), mesenchymal/desmoplastic (M), mucinous (Mu), and poorly differentiated (P). The dominant viable tumor pattern was used; no expression-guided ROI was selected. Initial image assignments were independently reviewed by a board-certified pathologist (T.S.), and disagreements were resolved by consensus. Reviewers did not use module scores, survival outcomes, CMS labels, or ascites culture results during morphology assignment. Formal inter-observer kappa could not be calculated retrospectively because separate reader-level calls were not retained. The classification is therefore used as a research framework for the stromal axis and is not presented as a validated diagnostic classifier. Overall survival was obtained from the TCGA Clinical Data Resource. The primary multivariable Cox model compared M-pattern with D-pattern tumors and adjusted for age, stage (I/II versus III/IV), primary site (COAD versus READ), MANTIS MSI status, and ABSOLUTE tumor purity. CMS subtype was added only in a sensitivity analysis because CMS partly contains the stromal signal under study. Hazard ratios, 95% confidence intervals, *p* values, and proportional hazards tests were reported.

For H&E-associated transcriptomic analyses, we performed one-versus-rest differential expression analysis for each histological pattern and calculated pattern reference scores from top-ranked upregulated genes. M-reference top-ranked genes were used to test whether desmoplastic/stromal morphology was reflected in bulk mRNA profiles and to compare the M axis with CMS subtypes. GSE39582 [38] served as an independent bulk validation cohort for recurrence-free survival. Spatially derived signatures and module scores were projected into GSE39582 without gene reselection or optimization using recurrence outcomes.

### 2.2. Spatial Transcriptomic Datasets and Section-Wise Analysis

HEST-1K CRC spatial transcriptomic data were obtained from MahmoodLab/hest on Hugging Face (version 1.3.0), the only spatial transcriptomic resource used in this study [36]. Whole-section H&E images were classified before transcriptomic scoring using the same binary end-point definitions: M-type denoted stroma-dominant, mesenchymal/desmoplastic morphology, and D-type denoted differentiated, gland-forming, cancer cell-dominant morphology. Initial assignments were independently reviewed by a board-certified pathologist (T.S.), with consensus resolution of disagreements. The reviewers were unaware of expression data, module scores, outcomes, CMS labels, and ascites culture results. No manual transcriptomic ROI was selected; each retained section was analyzed in its entirety. Of 22 CRC sections reviewed, two with ambiguous morphology were excluded before module projection, leaving 20 sections from 12 patient/tissue units. The M-type group comprised 10 sections from five units (TENX49; TENX89-92; ZEN36 and ZEN40; ZEN45; MISC66 and MISC67). The D-type group comprised 10 sections from seven units (ZEN39 and ZEN43; ZEN48 and ZEN49; ZEN38; MISC46 and MISC48; MISC43; MISC56; MISC70) (Supplementary Figure S1). Serial sections from the same specimen were averaged within the unit, and all primary spatial effect sizes used the unit as the independent observation (*n* = 5 versus 7). Formal kappa was unavailable because separate reader-level calls were not retained.

Candidate sections were screened for tissue and marker coverage, but final section inclusion and M-type/D-type assignment were based on the consensus H&E assessment described above. Predefined epithelial and stromal markers were used for compartment definition and quality control, not as the final morphological label. The co-authors did not use TCGA survival, CMS labels, GSE39582 recurrence outcomes, or ascites culture RNA-seq results to assign the HEST morphology groups. No formal batch correction or cross-dataset integration was applied to the spatial count matrices. The analysis used section-wise scoring, section-wise thresholds, and within-section neighborhood comparisons to avoid interpreting absolute cross-section expression differences as global differential expression.

No manual tumor ROI selection was applied in the HEST spatial analysis. After H&E-based section selection, analyses were performed across the whole tissue-covered section. EPCAM/KRT-high spots across each whole section were treated as cancer cell-enriched spots, whereas CAF/stroma-high spots across each whole section were used to define stromal-rich neighborhoods and proximity.

### 2.3. Compartment Scoring, Module Scoring, and Epithelial-Enriched Spots

Epithelial content and CAF/stroma content scores were calculated for each spatial spot using predefined epithelial and stromal marker sets. The epithelial content score used EPCAM and cytokeratin-associated genes, whereas the CAF/stroma content score used collagen, extracellular matrix, and fibroblast-associated genes. EPCAM/KRT-high spots were defined within each whole section as spots with epithelial content scores at or above the 60th percentile. CAF/stroma-high spots were defined within each whole section as spots with CAF/stroma content scores at or above the 80th percentile.

HEST was used to validate predefined CAF-coupled adaptive modules rather than to derive a new spatial signature. Compartment and module scores were projected onto HEST spots, and whole-section EPCAM/KRT-high contrasts between M-type and D-type sections were used to summarize directionality and nominate representative genes for visualization. The module definitions were fixed before projection into TCGA, GSE39582, single-cell datasets, and ascites-derived culture analyses.

All module gene sets were fixed before analysis and were not selected from the HEST M-type versus D-type contrast or from outcome data. The four primary modules represented EMT/pEMT, ECM/integrin, YAP/TAZ-TEAD mechanotransduction, and DTP/persister biology. The core adaptive composite was an operational summary calculated as the unweighted mean of these four module scores; it did not introduce additional genes and was not treated as an independently established biological pathway. EMT/pEMT genes were informed by the MSigDB Hallmark EMT set and published partial EMT literature [16,17,26]. ECM/integrin genes were informed by Reactome, GO, and KEGG integrin, ECM–receptor interaction, and focal adhesion sets. The YAP/TAZ-TEAD set used published mechanotransduction targets and regulators [27,28], including YAP1, WWTR1, TEAD-family genes, CCN1, CCN2, and ANKRD1. DTP/persister genes were informed by the studies of Sharma, Hangauer, and Rehman [30,31,32]. For each dataset, a module score was the mean z-normalized expression of available constituent genes. No cross-platform integrated expression space was created. Complete gene membership, gene counts, provenance, and scoring notes for all primary and control modules are provided in Supplementary Tables S1–S5. A sensitivity analysis in the ascites cultures repeated scoring after removal of canonical stromal/CAF genes; a second scoring algorithm was not substituted because the fixed mean-z approach was used to preserve comparability across analysis layers.

The primary HEST module analyses used EPCAM/KRT-high spots, with tumor core spots as a more restrictive epithelial-dense subset. To test whether canonical stromal transcripts retained within the module definitions accounted for the results, we repeated these same comparisons after excluding 31 canonical stromal/CAF-associated genes from the fixed module sets. We retained the same sections, EPCAM/KRT-high and tumor core definitions, 55-section q95 reference, normalization, thresholds, and unit-level statistical procedures. The same exclusion set was applied to the ascites culture analysis. This sensitivity analysis changed module membership only; it did not redefine the spatial compartments or imply complete biological removal of stromal contributions from spatial spots or bulk cultures. The exclusion list and retained module membership are provided in Supplementary Figure S7 and its legend.

### 2.4. CAF-Neighborhood, Proximity, and Ligand–Receptor Analyses

For local neighborhood analyses, a CAF neighbor score was calculated for each EPCAM/KRT-high spot as the mean CAF/stroma content score of the 12 nearest spatial spots within each section. EPCAM/KRT-high spots were divided into section-wise quintiles by CAF neighbor score, and module activity was compared between CAF neighbor-high and CAF neighbor-low spots. Mixed-effects models tested whether the association between CAF neighbor score and epithelial module activity differed between stromal-low and stromal-rich sections, with section modeled as a random effect.

For proximity analyses, the Euclidean distance from each EPCAM/KRT-high spot to the nearest CAF/stroma-high spot was calculated within each section. The closest and farthest section-wise quintiles were defined as CAF-proximal and CAF-distant spots. Because spatial spots can contain mixed transcripts, proximity analyses were interpreted as epithelial-enriched interface signals rather than proof of purely malignant epithelial expression.

Candidate ligand–receptor analyses were prespecified and interpreted as expression-inferred hypotheses. In annotated single-cell data, curated ligand–receptor pairs were scored only when the ligand and receptor were assigned to the specified sender and receiver cell classes. In HEST, direction labels denoted paired ligand and receptor gene-set axes measured in the relevant EPCAM/KRT-high and CAF/stroma compartments; these axis scores were not treated as evidence of a physical interaction between individual cells. The selection rationale, complete explicit single-cell pairs, and all HEST ligand and receptor axis members are listed in Supplementary Table S8. No ligand–receptor result was interpreted as proof of direct signaling or causality.

### 2.5. Bulk, Single-Cell, and Public Perturbation Projections

The CAF/stroma component, the cancer cell program component, the integrated composite niche score, and individual module scores were projected into TCGA and GSE39582. The cancer cell program component was the same operational mean of EMT/pEMT, ECM/integrin, YAP/TAZ-TEAD, and DTP/persister scores and excluded the CAF/stroma score. In GSE39582, recurrence-free survival was analyzed with multivariable Cox models adjusted for stage, tumor location, mismatch repair status, and adjuvant chemotherapy. Original module scores, already calculated as mean constituent gene z-scores, were entered directly as continuous variables without a second standardization step. A prespecified upper tertile versus lower-two-thirds comparison was used for adjusted sensitivity models and descriptive Kaplan–Meier curves; no outcome-optimized cutoff was used. Hazard ratios, 95% confidence intervals, *p* values, BH-adjusted q values, and proportional hazards tests are reported in Supplementary Table S7.

Public CRC single-cell RNA-seq datasets GSE132465 and GSE144735 were used to evaluate the fixed modules within annotated cancer and stromal cells [39]. The core composite, defined as the unweighted mean of EMT/pEMT, ECM/integrin, YAP/TAZ-TEAD, and DTP/persister scores, was used to stratify cancer cells as core-composite-high, intermediate, or core-composite-low. The CAF/stroma module was not included in this cancer cell score. CAF/stromal fractions were then compared across samples enriched for core-composite-high or core-composite-low cancer cells.

Public CAF co-culture perturbation datasets GSE214569 [33] and GSE198697 [34] were analyzed as orthogonal perturbation datasets. Module effects were summarized using Cohen’s d for CAF-exposed versus control conditions.

### 2.6. Ascites-Derived Primary Culture RNA-Seq and Primary Tumor Morphology Grouping

Patient-derived malignant ascites cultures were used to evaluate whether EMT/adaptive programs remained associated with primary stromal architecture after ex vivo propagation. Baseline/control bulk RNA-seq profiles from ascites-derived primary cultures were analyzed. Primary tumor histology was reviewed and grouped into stroma-dominant (M-type) and differentiated cancer cell-dominant (D-type) patterns. Four M-type-derived cultures and an equal number of D-type-derived cultures with matched primary tumor H&E specimens and baseline RNA-seq data were included. Selection was performed without reference to module scores or transcriptomic results. The overall culture establishment success rate was 71.4%. Module scoring in ascites-derived cultures evaluated morphology-associated cancer cell programs. The eight cultures were maintained for 1–2.5 months and sampled at passages 3–7; case-level passage, collection timing, primary site, metastatic site, molecular status, and prior treatment are provided in Table 2. The cultures were enriched through sustained ex vivo expansion of adherent tumor cells but were not purified by FACS, and formal tumor purity estimates were unavailable. We also recalculated the four primary modules after excluding canonical stromal/CAF genes, including collagens, FN1, DCN, LUM, PDGFRB, ACTA2, FAP, POSTN, SPARC, TNC, and VCAN. Because these were bulk culture profiles, we refer to culture-level or cancer cell-associated programs rather than purified malignant cell-intrinsic states. In the same baseline cultures, curated ligand and receptor gene-set axes were summarized as expression-inferred candidate CAF-to-cancer or cancer-to-CAF programs. Because bulk RNA-seq does not establish the cellular source of either component, the direction labels indicate the prespecified axis model and are not evidence of direct cell–cell communication. The ascites culture analysis comprised eight independent patient-derived cultures, including four derived from M-type tumors and four derived from D-type tumors. The cohort size was determined by the availability of matched primary tumor H&E morphology and baseline RNA-seq data. This analysis was therefore considered exploratory.

### 2.7. CAF Co-Culture and VIM Promoter Reporter Assay

To test whether CAF exposure could increase EMT-like output in cancer cells, RKO and SW480 cells expressing a VIM promoter-driven RFP reporter [40] were cultured with or without GFP-expressing CAFs. Red-positive area and integrated red signal intensity were quantified per region of interest and compared between monoculture and CAF co-culture. RKO cells were obtained from the American Type Culture Collection (ATCC). SW480 cells were originally obtained from the Cell Resource Center for Biomedical Research, Institute of Development, Aging and Cancer, Tohoku University (Sendai, Japan), and were subsequently maintained as laboratory stocks.

### 2.8. Statistics, Ethics, and Data Availability

All statistical analyses were performed in R unless otherwise stated. Group differences were summarized with Cohen’s d and Wilcoxon, exact permutation, or *t*-tests as specified for each analysis. BH correction was applied separately within prespecified analysis families: spatial module comparisons, CAF-neighborhood comparisons, ligand/receptor-axis comparisons, ascites culture module comparisons, and survival module models. The primary spatial outcomes were the four fixed module scores and their operational composite in EPCAM/KRT-high spots at the patient/tissue unit level. TCGA overall survival and GSE39582 recurrence-free survival were the prespecified clinical endpoints. Kaplan–Meier plots were descriptive; adjusted Cox models provided the primary survival inference. Reported *p* values were two-sided. The use of residual specimens was approved by the Institutional Review Board of Kawasaki Medical School (approval no. 6281; approved on 4 January 2024). Written consent for routine pathological examination and oral consent for research use were obtained in accordance with the approved protocol.

Public data sources, versions, sample identifiers, and access dates are specified in the Data Availability Statement. HEST section identifiers and patient/tissue-unit mapping are provided in Supplementary Table S1.

### 2.9. Declaration of Generative AI and AI-Assisted Technologies in the Manuscript Preparation Process

During the preparation of this work, the authors used ChatGPT (GPT-5.5, OpenAI, San Francisco, CA, USA) to improve the readability and language of the manuscript. Draft sections originally written in the authors’ native language (Japanese) were translated and refined into English with the assistance of this tool. ChatGPT was also used to support the development of data analysis code. All AI-assisted code was reviewed, tested, and verified by the authors before use. The authors carefully reviewed and edited all AI-assisted output as needed and take full responsibility for the content of this publication.

## 3. Results

### 3.1. H&E Morphology Defines a Prognostic Stromal CRC Axis

**Figure 1:**
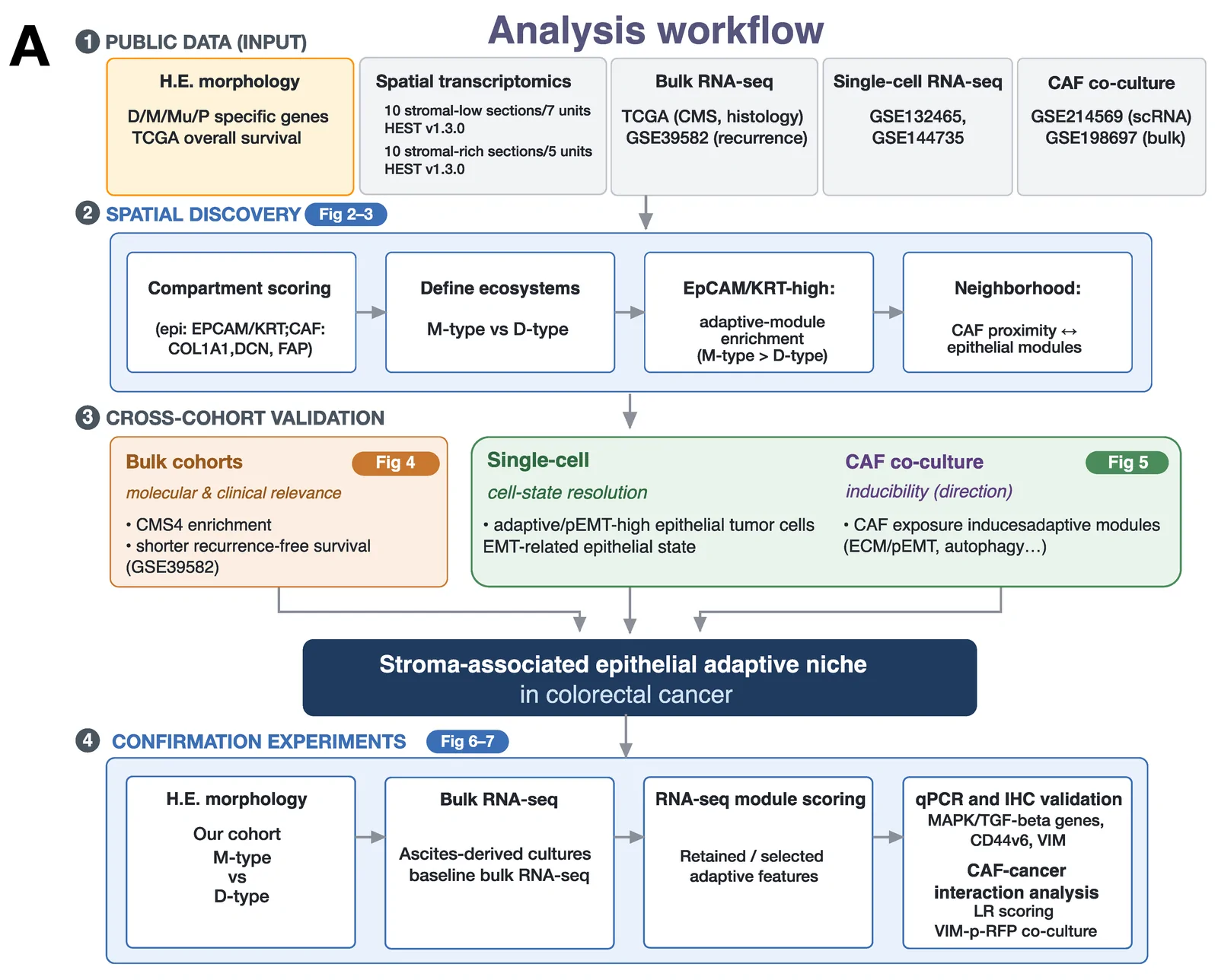

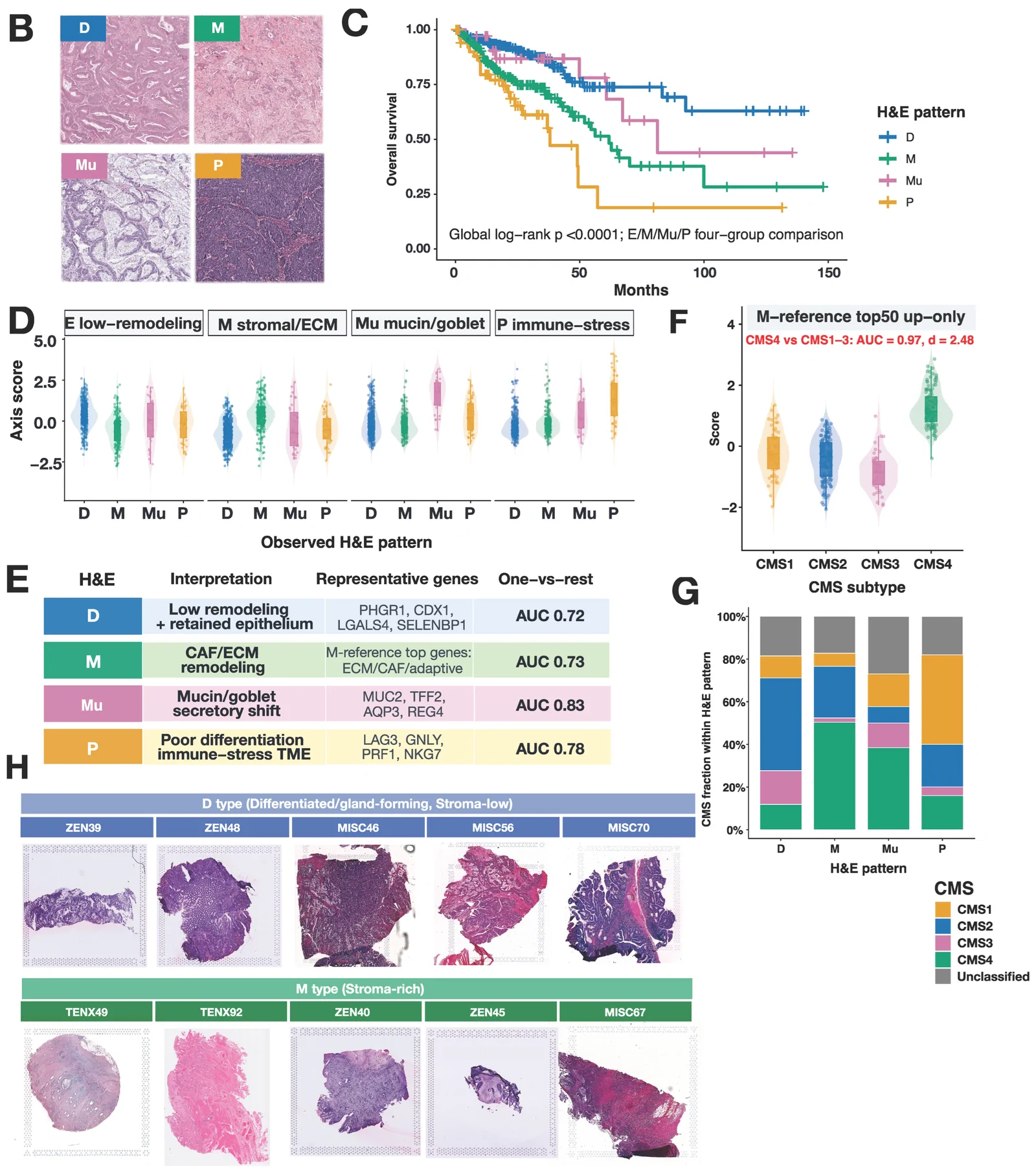
H&E morphology defines prognostic CRC patterns and a CMS4-like stromal axis. (**A**) Analysis workflow integrating TCGA bulk RNA-seq, H&E histology, CMS labels, HEST-1K spatial transcriptomics, single-cell atlases, and CAF co-culture datasets. (**B**) Representative H&E images of the four histological patterns: differentiated/gland-forming (**D**), mesenchymal/desmoplastic (M), mucinous (Mu), and poorly differentiated (P). (**C**) Overall survival in TCGA CRC stratified by H&E pattern (*n* = 597; events = 124; global log-rank *p* < 0.0001). (**D**) Pattern-specific transcriptomic axis scores across H&E patterns. (**E**) Summary of H&E pattern interpretation with representative genes and one-versus-rest classification performance (AUC). (**F**) M-reference top-50 upregulated gene scores across CMS subtypes. (**G**) CMS subtype composition within each H&E pattern. (**H**) Representative HEST-1K CRC sections from the M-type (stroma-rich) and D-type (stroma-low) groups.

TCGA CRC H&E annotations, CMS labels, bulk RNA-seq profiles, and clinical outcomes provided the initial morphology-to-molecular framework (Figure 1A). Diagnostic H&E images were assigned to four prespecified categories: differentiated/gland-forming (D), mesenchymal/desmoplastic (M), mucinous (Mu), and poorly differentiated (P) (Figure 1B). In the survival-annotated cohort (*n* = 597; 124 deaths), overall survival differed across the four groups (global log-rank *p* < 0.0001; Figure 1C): D-pattern tumors had the most favorable course, M- and P-pattern tumors the poorest, and Mu-pattern tumors an intermediate course.

In the complete-case multivariable analysis (*n* = 426; 77 deaths), M-pattern morphology remained associated with poorer overall survival after adjustment for age, stage, primary site, MANTIS MSI status, and ABSOLUTE tumor purity (HR = 2.94, 95% CI 1.75–4.95; *p* < 0.001; Table 1). The association was similar after CMS was added (*n* = 275; 58 deaths; HR = 2.41, 95% CI 1.28–4.55; *p* = 0.0066). In models containing both morphology and one continuous module score, morphology remained significant while the module term added no independent overall survival information. 

M-pattern morphology therefore carried integrated prognostic information shared with, but not reducible to, the individual transcriptomic modules. The proportional hazards assumption was met.

Bulk RNA-seq reflected these visual categories at the molecular level. Pattern-specific transcriptomic axis scores separated the four H&E groups: D was associated with retained epithelial differentiation (PHGR1, CDX1, LGALS4), M with stromal/ECM remodeling, Mu with mucin/goblet secretory differentiation (MUC2, TFF2, REG4), and P with poor differentiation and immune stress programs (LAG3, GNLY, PRF1) (Figure 1D,E). In one-versus-rest classification, each axis discriminated its own pattern (AUC 0.72–0.83; Figure 1E), confirming that the visual patterns correspond to distinct transcriptional states. Because M morphology captured a stromal/ECM-remodeling program, we next asked how it related to the consensus molecular subtypes. An M-reference top-50 upregulated gene score strongly separated CMS4 from CMS1–3 tumors (AUC = 0.97; Cohen’s d = 2.48; Figure 1F), indicating that H&E M morphology and the mesenchymal CMS4 subtype share a common stromal axis. However, the two were not interchangeable: although CMS4 was enriched within M-pattern tumors, a substantial CMS4 fraction was distributed across D, Mu, and P categories, and M-pattern tumors contained non-CMS4 cases (Figure 1G). This partial overlap indicated that neither H&E M-type nor CMS4 fully captures the underlying stroma-associated biology, motivating a spatially resolved dissection of the stromal axis.

The incomplete overlap among morphology, CMS4, and prognosis led us to examine the stromal axis at spatial resolution. HEST section assignment preceded all transcriptomic analysis. Initial H&E assignments were independently reviewed by a board-certified pathologist without access to expression data or clinical outcomes, and two of 22 ambiguous sections were excluded before scoring. The remaining 20 sections represented 12 independent patient/tissue units: five M-type, stroma-dominant units and seven D-type, differentiated cancer cell-dominant units (Figure 1H).

### 3.2. Cancer Cell-Enriched Spatial Spots Reveal EMT, Matrix-Engagement, and Survival Programs in M-Type CRC

We restricted module analysis to EPCAM/KRT-high spots to reduce stromal admixture while retaining cancer cell-containing regions. All effect sizes used the patient/tissue unit as the independent observation. The four primary gene sets were fixed before HEST projection. For compact presentation, their unweighted mean is shown as the core adaptive composite; this is an operational summary of EMT/pEMT, ECM/integrin, YAP/TAZ-TEAD, and DTP/persister scores rather than a separate pathway.

**Figure 2:**
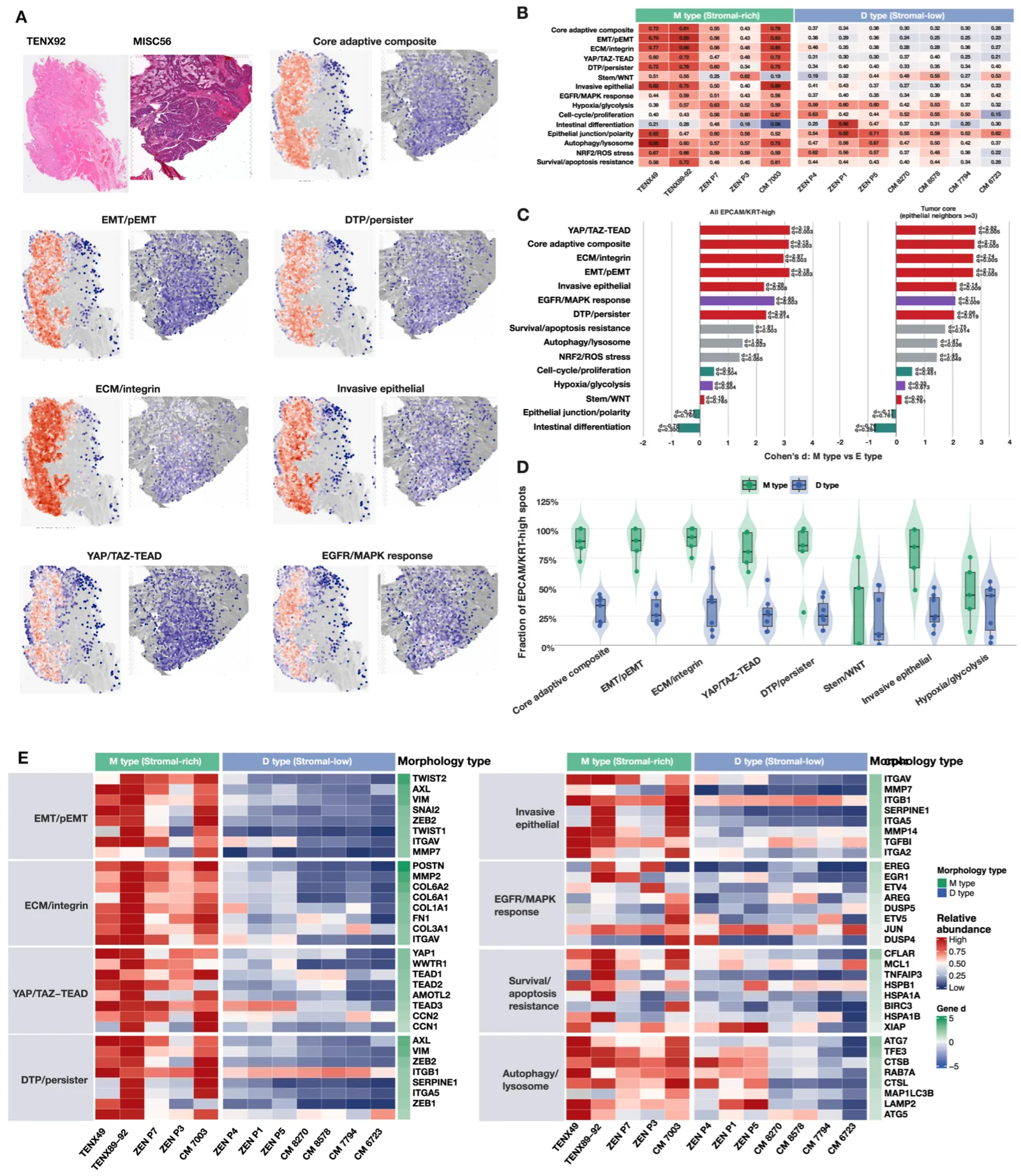
Epithelial-enriched spatial spots reveal CAF-associated adaptive programs in M-type colorectal cancer sections. Analyses use HEST-1K sections classified by H&E morphology as mesenchymal/desmoplastic (M-type, stroma-rich; 10 sections, 5 patient/tissue units) or well-differentiated/gland-forming (D-type, stroma-low; 10 sections, 7 units). All effect sizes and comparisons are computed at the unit level (*n* = 5 M-type vs. 7 D-type). (**A**) Representative M-type (TENX92) and D-type (MISC56) sections. H&E images in the remaining panels show module activity projected onto EPCAM/KRT-high spots for the core adaptive composite, EMT/pEMT, DTP/persister, ECM/integrin, invasive epithelial, YAP/TAZ-TEAD, and EGFR/MAPK response modules, with the M-type section on the left and the D-type section on the right within each pair. Warm colors indicate high and cool colors indicate low module activity. The core adaptive composite is the mean of the EMT/pEMT, ECM/integrin, YAP/TAZ-TEAD, and DTP/persister modules. (**B**) Per-unit module score matrix across the 12 units (5 M-type, 7 D-type). Cell values are mean module scores per unit. (**C**) Effect sizes (Cohen’s d, M-type versus D-type) at the unit level. Left, all EPCAM/KRT-high spots; right, tumor core spots (EPCAM/KRT-high spots with at least three epithelial neighbors, minimal stromal admixture). Core adaptive modules remained large in tumor core spots (d > 2, q ≤ 0.02), arguing against a simple spillover-only explanation while not excluding residual admixture or section-level confounding. Bars are colored by program category; Benjamini–Hochberg q values are shown beside each bar. (**D**) Fraction of EPCAM/KRT-high spots above the D-type reference threshold for each module in M-type (green) and D-type (blue) units. Points are individual units; violins show the distribution and boxes the median and interquartile range. (**E**) Gene-level heatmap of module constituents in EPCAM/KRT-high spots across the 12 units, grouped by module. Cell values are relative abundance; the side annotation shows the per-gene effect size (M-type versus D-type). In the spatial maps (**A**) and heatmaps (**B**,**E**), warm red colors indicate higher relative module or gene abundance, cool blue colors indicate lower abundance, and pale colors indicate intermediate values.

In a representative M-type (TENX92) and D-type (MISC56) section, the core adaptive composite, EMT/pEMT, ECM/integrin, YAP/TAZ-TEAD, DTP/persister, and invasive epithelial modules were broadly and coherently active across the cancer cell compartment of the M-type section, whereas the D-type section showed only limited or focal activity (Figure 2A).

Per-unit module scores confirmed this separation across all 12 units (Figure 2B). The core adaptive composite, EMT/pEMT, ECM/integrin, YAP/TAZ-TEAD, and DTP/persister modules were consistently higher in the five M-type units than in the seven D-type units, with minimal overlap. Reference programs including hypoxia/glycolysis, cell cycle/proliferation, epithelial junction/polarity, and intestinal differentiation showed no such separation and were similar to or higher than in D-type units.

Quantifying the M-versus-D contrast across all EPCAM/KRT-high spots, the largest effect sizes were seen for YAP/TAZ-TEAD (Cohen’s d = 3.19), EMT/pEMT (d = 3.18), the core adaptive composite (d = 3.15), and ECM/integrin (d = 2.97) (all q = 0.00316), followed by EGFR/MAPK response (d = 2.65, q = 0.003), DTP/persister (d = 2.35, q = 0.0142), and invasive epithelial (d = 2.28, q = 0.00812) (Figure 2C, left). Stress survival programs separated the groups more modestly (survival/apoptosis resistance d = 1.91, autophagy/lysosome d = 1.52, NRF2/ROS stress d = 1.41). Reference programs not central to the adaptive axis (cell cycle/proliferation, hypoxia/glycolysis, stem/WNT, epithelial junction/polarity, and intestinal differentiation) showed no separation (|d| ≤ 0.75, all q > 0.3), with intestinal differentiation trending higher in D-type sections (d = −0.78).

We repeated the analysis in tumor core spots, defined as EPCAM/KRT-high spots with at least three epithelial neighbors. M-type versus D-type differences remained large for YAP/TAZ-TEAD (d = 2.82), the core adaptive composite (d = 2.78), ECM/integrin (d = 2.74), and EMT/pEMT (d = 2.73; all q = 0.005), as well as invasive epithelial (d = 2.14), EGFR/MAPK response (d = 2.11), and DTP/persister (d = 2.06) (Figure 2C, right). Effects of this magnitude in regions with minimal stromal content place a substantial part of the signal within the cancer cell-enriched compartment.

Using the same EPCAM/KRT-high and tumor core spot definitions, we then tested whether canonical stromal transcripts within the module sets accounted for the observed differences (Supplementary Figure S7A). After excluding the same 31 canonical stromal/CAF-associated genes, M-type effects across all EPCAM/KRT-high spots remained for YAP/TAZ-TEAD (d = 3.19, q = 0.004), EMT/pEMT (d = 3.18, q = 0.004), the core adaptive composite (d = 2.85, q = 0.004), DTP/persister (d = 2.35, q = 0.014), invasive epithelial (d = 2.28, q = 0.009), and the filtered ECM/integrin module (d = 1.96, q = 0.014) (Supplementary Figure S7B–D,F; left). The pattern also remained in tumor core spots, including YAP/TAZ-TEAD (d = 2.82, q = 0.006), EMT/pEMT (d = 2.73, q = 0.006), the core adaptive composite (d = 2.54, q = 0.006), DTP/persister (d = 2.06, q = 0.022), invasive epithelial (d = 2.14, q = 0.011), and filtered ECM/integrin (d = 1.84, q = 0.026) (Supplementary Figure S7C,F; right). Thus, the added analysis tested dependence on module membership rather than applying a new epithelial spot restriction.

In M-type sections, roughly 80–95% of EPCAM/KRT-high spots scored positive for the core adaptive composite, EMT/pEMT, ECM/integrin, YAP/TAZ-TEAD, and DTP/persister modules, compared with approximately 25–45% in D-type sections, with little overlap; reference programs such as stem/WNT and hypoxia/glycolysis overlapped between the two groups (Figure 2D).

Gene-level heatmaps reproduced the contrast across the individual constituents of each module (Figure 2E). EMT/pEMT (TWIST2, AXL, VIM, SNAI2, ZEB2, TWIST1, ITGAV, MMP7), ECM/integrin (POSTN, MMP2, COL6A2, COL6A1, COL1A1, FN1, COL3A1, ITGAV), YAP/TAZ-TEAD (YAP1, WWTR1, TEAD1-3, AMOTL2, CCN1, CCN2), DTP/persister (AXL, VIM, ZEB2, ITGB1, SERPINE1, ITGA5, ZEB1, CD44), and invasive epithelial (ITGAV, MMP7, ITGB1, SERPINE1, ITGA5, MMP14, TGFBI, ITGA2) genes were coordinately higher in M-type units, with concordant elevation of EGFR/MAPK response, survival/apoptosis resistance, and autophagy/lysosome constituents, confirming that the module-level differences reflect multi-gene programs rather than individual genes (Supplementary Figure S2).

### 3.3. Cancer Cell Programs Are Spatially Coupled to a Bidirectional CAF Niche

**Figure 3:**
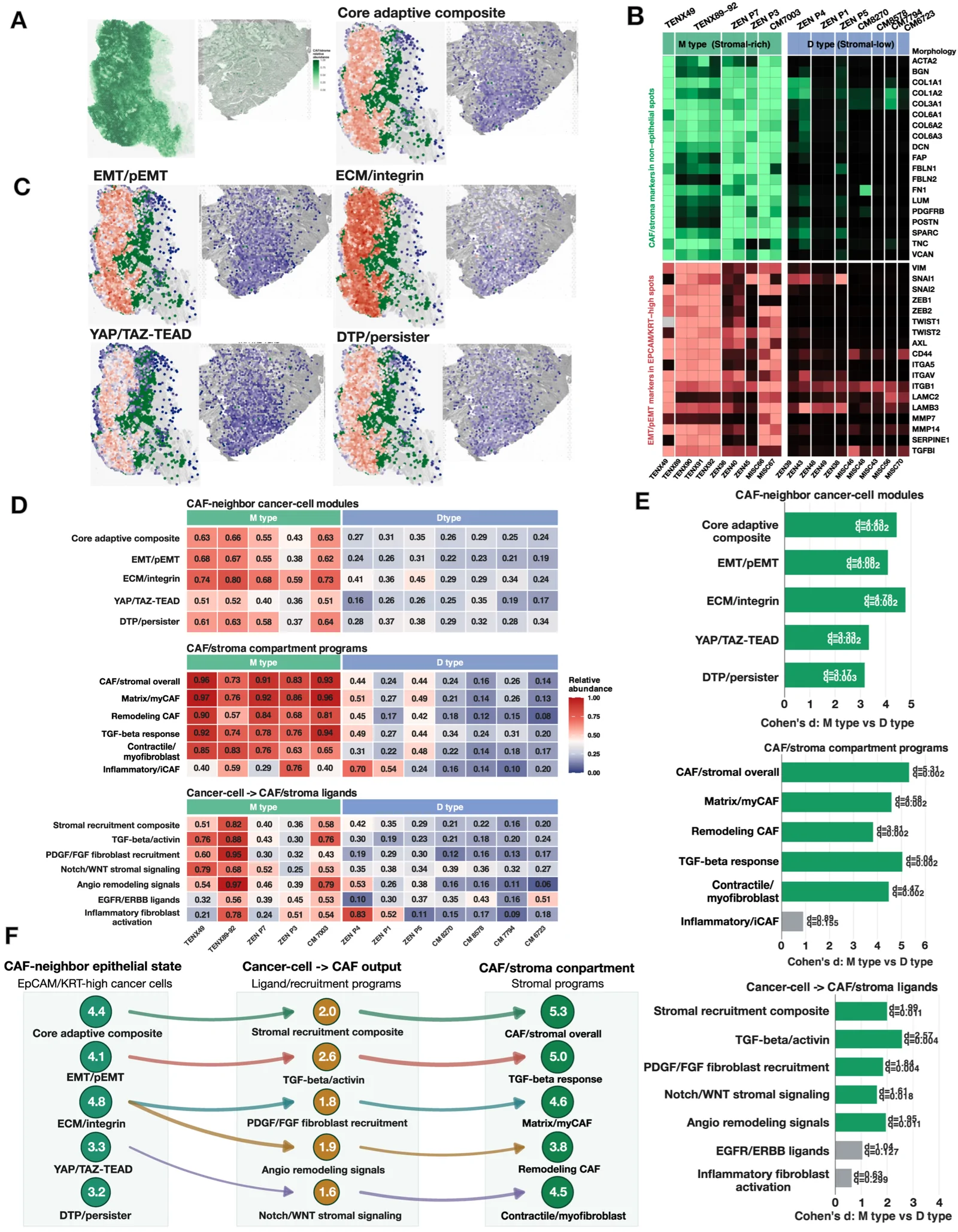
Cancer cell programs are spatially coupled to a bidirectional CAF niche in M-type colorectal cancer. All comparisons use HEST-1K sections classified by H&E morphology as M-type (stroma-rich; 5 patient/tissue units) or D-type (stroma-low; 7 units), with effect sizes computed at the unit level (M-type versus D-type). (**A**) CAF/stroma compartment, scored in non-epithelial spots, in a representative M-type (TENX92) and D-type (MISC56) section. Green intensity indicates CAF marker abundance. (**B**) Gene-level heatmaps across the 12 units. Top, CAF/stroma markers in non-epithelial spots (green); bottom, EMT/pEMT markers in EPCAM/KRT-high spots (red). The upper annotation bar indicates M-type and D-type morphology. Color scales are fixed to the cross-unit reference. (**C**) EPCAM/KRT-high spots overlaid on the CAF/stroma compartment (green) and colored by module activity in the representative M-type and D-type sections, for the core adaptive composite, EMT/pEMT, ECM/integrin, YAP/TAZ-TEAD, and DTP/persister modules, with the M-type section at left and the D-type section at right within each pair. Adaptive-module activity concentrates at the CAF-facing epithelial interface in the M-type section. The core adaptive composite is the mean of the EMT/pEMT, ECM/integrin, YAP/TAZ-TEAD, and DTP/persister modules. (**D**) Per-unit score matrices for three program sets: CAF neighbor cancer cell modules in EPCAM/KRT-high spots (top), CAF/stroma compartment programs in non-epithelial spots (middle), and cancer cell-to-CAF ligand/recruitment programs in EPCAM/KRT-high spots (bottom). Columns are the 12 units (5 M-type, 7 D-type); cell values are mean scores per unit. (**E**) Effect sizes (Cohen’s d, M-type versus D-type) for the three program sets in (**D**). Benjamini–Hochberg q values are indicated; gray bars denote programs that did not separate the groups (inflammatory/iCAF; EGFR/ERBB ligands; inflammatory fibroblast activation). (**F**) Summary of the bidirectional niche. Left, CAF neighbor cancer cell program state; middle, cancer cell-to-CAF ligand/recruitment output; right, CAF/stroma compartment programs. Values are unit-level effect sizes (Cohen’s d, M-type versus D-type). In the spatial maps (**A**,**C**), darker green indicates higher CAF-marker abundance; in (**C**), green identifies the CAF/stroma compartment, whereas warm red and cool blue indicate higher and lower module activity, respectively, in EPCAM/KRT-high spots. In the heatmaps, brighter green or red indicates higher and black indicates lower marker abundance in (**B**), whereas red, blue, and pale colors indicate higher, lower, and intermediate relative program abundance, respectively, in (**D**).

We then examined whether the section-wide cancer cell program differences in M-type sections localized to CAF-rich niches. In a representative M-type section (TENX92), non-epithelial spots formed a broad, contiguous CAF/stroma territory; the D-type section (MISC56) contained only sparse foci (Figure 3A). Across all 12 units, gene-level heatmaps showed higher canonical CAF/stroma markers (ACTA2, FAP, PDGFRB, POSTN, COL1A1, COL3A1, FN1, VCAN) in non-epithelial spots and higher EMT/pEMT markers (VIM, ZEB2, TWIST2, AXL, ITGAV, ITGB1, SERPINE1, TGFBI) in EPCAM/KRT-high spots from M-type units (Figure 3B; Supplementary Figure S3). In the representative M-type section, the core adaptive composite, EMT/pEMT, ECM/integrin, YAP/TAZ-TEAD, and DTP/persister programs concentrated at the CAF-facing epithelial interface. The corresponding interface was sparse and showed little activity in the D-type section (Figure 3C; Supplementary Figure S4).

Quantifying CAF neighbor cancer cell modules at the unit level, every core adaptive module was markedly higher in M-type than in D-type units: ECM/integrin (Cohen’s d = 4.78), the core adaptive composite (d = 4.43), EMT/pEMT (d = 4.08), YAP/TAZ-TEAD (d = 3.33), and DTP/persister (d = 3.17) (all q ≤ 0.003) (Figure 3D, top; Figure 3E, left; Figure 3F, left). Per-unit scores were consistently higher in the five M-type units (approximately 0.4–0.8) than in the seven D-type units (approximately 0.2–0.45), with minimal overlap (Figure 3D, top; Supplementary Figure S4). Increasing numbers of CAF-high neighboring spots were also associated with higher cancer cell module activity across most independent units (Supplementary Figure S5).

The stromal compartment showed a matched program in the same M-type units. Overall CAF/stroma content (d = 5.31), TGF-beta response (d = 5.04), matrix/myCAF (d = 4.58), contractile/myofibroblast (d = 4.47), and remodeling CAF (d = 3.81) separated M-type from D-type units (all q = 0.002), whereas inflammatory/iCAF did not (d = 0.89, q = 0.155) (Figure 3D, bottom; Figure 3E, right; Figure 3F, middle; Supplementary Figure S3). Matrix remodeling and myofibroblast programs dominated the expanded stromal compartment in M-type CRC.

M-type epithelial regions expressed a selective set of candidate ligands directed toward the stroma (Figure 3D, bottom; Figure 3E, right; Figure 3F, middle). TGF-beta/activin (d = 2.57, q = 0.004), the composite stromal recruitment program (d = 1.99, q = 0.011), angio-remodeling (d = 1.95, q = 0.011), PDGF/FGF fibroblast recruitment (d = 1.84, q = 0.004), and Notch/WNT stromal signaling (d = 1.61, q = 0.018) were elevated. EGFR/ERBB ligands (d = 1.04, q = 0.127) and inflammatory fibroblast activation (d = 0.63, q = 0.299) were similar between groups. This selective output linked the matrix-remodeling CAF compartment to cancer cell programs centered on matrix engagement and TGF-beta signaling (Figure 3F).

### 3.4. Bulk and Single-Cell Cohorts Support a CAF-Associated Cancer Cell Program

**Figure 4:**
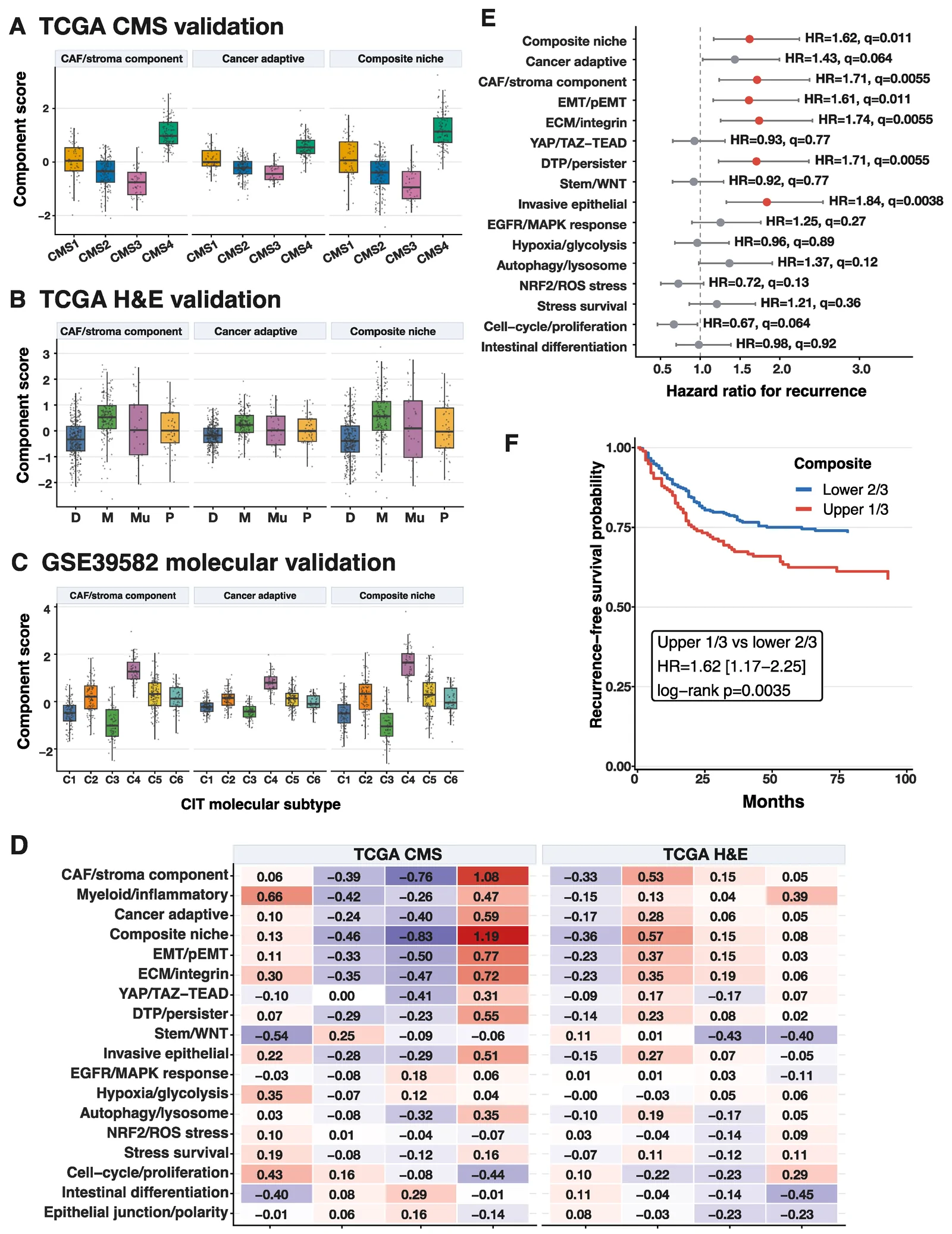
Bulk cohorts support conservation of the CAF-associated adaptive niche. (**A**) Component scores across TCGA CMS subtypes for the CAF/stroma component, cancer cell program component (epithelial modules, excluding CAF/stroma content), and integrated composite niche score. (**B**) Component scores across H&E-defined TCGA histological patterns. (**C**) Projection into GSE39582 across CIT molecular subtypes. (**D**) Module- and component-level heatmap across TCGA CMS subtypes (left) and H&E patterns (right). Cell values are mean scores. (**E**) Adjusted recurrence-free survival analysis in GSE39582 using the prespecified upper tertile versus lower-two-thirds comparison. Points show adjusted hazard ratios and bars the 95% confidence intervals. Models were adjusted for stage, tumor location, MMR status, and adjuvant chemotherapy. Invasive epithelial (HR = 1.84, q = 0.0017), ECM/integrin (HR = 1.89, q = 0.0017), CAF/stroma (HR = 1.75, q = 0.0025), DTP/persister (HR = 1.75, q = 0.0025), composite niche (HR = 1.67, q = 0.0046), and EMT/pEMT (HR = 1.68, q = 0.0046) were associated with recurrence; YAP/TAZ-TEAD was not (HR = 0.92, q = 0.660). (**F**) Kaplan–Meier curve for recurrence-free survival in GSE39582, stratified by the composite niche score (upper tertile versus lower two tertiles). HR = 1.62 (95% CI 1.17–2.25); log-rank *p* = 0.0035. In the spatial maps warm red colors indicate higher relative module or gene abundance, cool blue colors indicate lower abundance, and pale colors indicate intermediate values.

We projected the spatially defined niche into independent bulk cohorts as three component-level scores: CAF/stroma, the cancer cell program component excluding CAF/stroma content, and an integrated composite niche score. All three were highest in CMS4 and lowest in CMS3 tumors in TCGA CRC (Figure 4A), highest in H&E-defined M-pattern tumors (Figure 4B), and enriched in the stromal/mesenchymal CIT-C4 subtype in GSE39582 (Figure 4C).

Module-level heatmaps confirmed that the niche reflected coordinated stromal remodeling and epithelial plasticity rather than a single marker (Figure 4D). In CMS4, the composite niche (mean score 1.19) and CAF/stroma component (1.08) were most enriched, followed by EMT/pEMT (0.77) and ECM/integrin (0.72), whereas intestinal differentiation and cell cycle/proliferation were not enriched (−0.01 and −0.44). The same directionality was reproduced across H&E patterns, with the composite niche (0.57), CAF/stroma (0.53), EMT/pEMT (0.37), and ECM/integrin (0.35) highest in M-pattern tumors and lowest in D-pattern tumors.

The upper tertile of the composite niche score had shorter recurrence-free survival than the lower two tertiles (unadjusted HR = 1.62, 95% CI 1.17–2.25; log-rank *p* = 0.0035; Figure 4F). Covariate-adjusted analyses using the same prespecified strata confirmed shorter recurrence-free survival for the composite niche, EMT/pEMT, ECM/integrin, DTP/persister, and invasive epithelial scores, whereas YAP/TAZ-TEAD was not associated with recurrence (Supplementary Figure S8). Multivariable Cox models then entered the original mean gene-z scores and adjusted for stage, tumor location, mismatch repair status, and adjuvant chemotherapy (*n* = 492; 140 recurrence events). Recurrence hazards increased with EMT/pEMT (HR = 1.59, 95% CI 1.23–2.06; q = 0.0012), ECM/integrin (HR = 1.56, 95% CI 1.21–2.03; q = 0.0012), DTP/persister (HR = 1.83, 95% CI 1.30–2.58; q = 0.0012), invasive epithelial activity (HR = 1.78, 95% CI 1.35–2.36; q < 0.001), and the composite niche score (HR = 1.31, 95% CI 1.11–1.55; q = 0.0023). YAP/TAZ-TEAD was not associated with recurrence (HR = 1.35, 95% CI 0.93–1.96; q = 0.119). All primary models met the proportional hazards assumption. Complete multivariable Cox model results are provided in Table 1.

### 3.5. Single-Cell and Perturbation Analyses Link the Four-Program Cancer Cell State to CAF Abundance

The discordant YAP/TAZ-TEAD result separated spatial activity from prognostic association: the module distinguished M-type from D-type tissue but did not predict recurrence in GSE39582. Prognostic information was concentrated in EMT/pEMT, ECM/integrin, DTP/persister, invasive epithelial activity, and the composite niche score.

**Figure 5:**
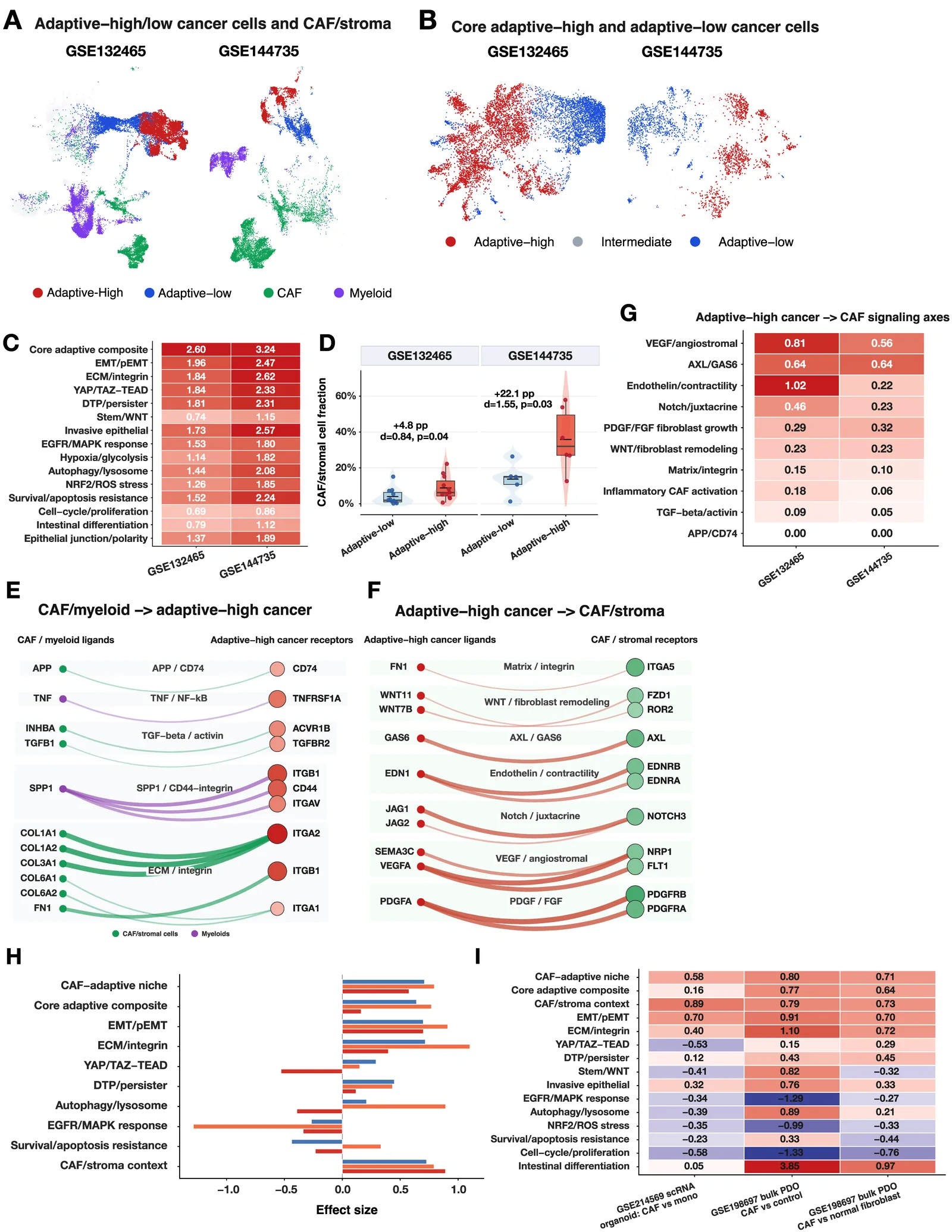
Single-cell and perturbation analyses support a candidate CAF-associated cancer cell program in CRC. (**A**) UMAPs of the CRC single-cell atlases GSE132465 and GSE144735, showing core-composite-high cancer cells, core-composite-low cancer cells, CAFs, and myeloid cells. Cancer cells were scored with the operational core-composite score, defined as the mean of the four fixed primary modules. (**B**) UMAPs restricted to cancer cells, showing core-composite-high, intermediate, and core-composite-low groups. (**C**) Module profile of core-composite-high cancer cells in each dataset. Cell values are effect sizes relative to other cancer cells. (**D**) CAF/stromal cell fraction in samples enriched for core-composite-high cancer cells versus samples dominated by core-composite-low cancer cells. Effect sizes and *p* values are shown. (**E**) Expression-inferred candidate ligand–receptor pairs from CAF/stromal and myeloid cells toward core-composite-high cancer cells, grouped by axis. Node size indicates the receptor delta z; edges are colored by annotated sender cell type. (**F**) Reverse expression-inferred analysis from core-composite-high cancer cells toward CAF/stromal receptors, grouped by axis. Node size indicates the CAF receptor mean z. (**G**) Summary of cancer-to-CAF signaling axes in both datasets. Cell values are interaction scores. (**H**) Effect sizes of CAF exposure in three public co-culture perturbation datasets: GSE214569 scRNA organoid (CAF versus monoculture) and GSE198697 bulk PDO (CAF versus control; CAF versus normal fibroblast). (**I**) Module-level heatmap of CAF co-culture effects across the same three comparisons. Cell values are effect sizes. In the spatial maps (**A**) and heatmaps (**C**,**G**,**I**), warm red colors indicate higher relative module or gene abundance, cool blue colors indicate lower abundance, and pale colors indicate intermediate values.

We next applied the fixed modules to CRC single-cell atlases GSE132465 and GSE144735, where cancer and stromal cells had separate annotations. The operational four-module composite stratified cancer cells into core-composite-high, intermediate, and core-composite-low groups (Figure 5A–C). Relative to other cancer cells, core-composite-high cells showed higher ECM/integrin, EMT/pEMT, YAP/TAZ-TEAD, and DTP/persister scores, while cell cycle/proliferation showed the smallest difference. At the sample level, enrichment of core-composite-high cancer cells accompanied a higher CAF/stromal fraction in GSE132465 (+4.8 percentage points; d = 0.84, *p* = 0.04) and GSE144735 (+22.1 points; d = 1.55, *p* = 0.03) (Figure 5D).

Expression-inferred ligand–receptor scoring identified coexpressed programs across annotated stromal and cancer cell populations. Candidate CAF/myeloid-to-cancer axes included ECM/integrin, SPP1/CD44–integrin, TGF-beta/activin, TNF/NF-kappaB, and APP/CD74 (Figure 5E). Candidate cancer-to-CAF axes included matrix/integrin, WNT/fibroblast remodeling, AXL/GAS6, endothelin/contractility, Notch, VEGF/angiostromal, and PDGF/FGF programs (Figure 5F,G). These axes define specific candidates for functional testing.

Public co-culture datasets provided a separate test of CAF responsiveness (Figure 5H,I). Across three comparisons, comprising GSE214569 single-cell organoid data and two GSE198697 bulk PDO contrasts, CAF exposure increased the CAF-adaptive niche score (effect size 0.58 to 0.80), EMT/pEMT (0.70 to 0.91), and ECM/integrin (0.40 to 1.10), while cell cycle/proliferation decreased (−0.58 to −1.33).

### 3.6. Ascites-Derived Cultures Show Selected Morphology-Associated Cancer Cell Programs

**Figure 6:**
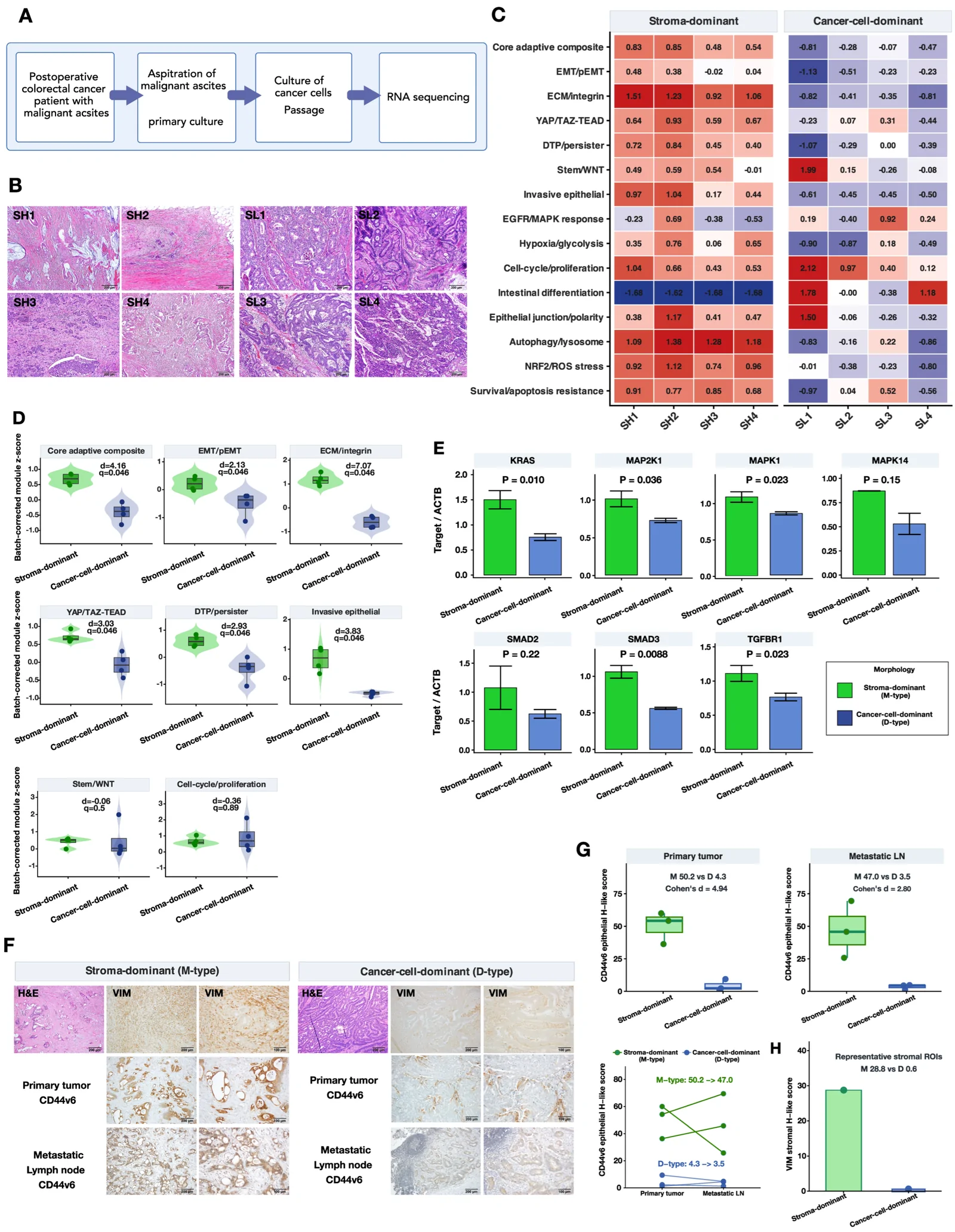
Morphology-associated adaptive programs in ascites-derived cultures and orthogonal validation by real-time PCR and IHC. (**A**) Schematic overview of malignant ascites collection, ascites-derived cancer cell-enriched culture, passaging, RNA sequencing, focused real-time PCR, and IHC validation. (**B**) Representative H&E images of primary tumors corresponding to stroma-dominant ascites cultures (SH1-SH4) and cancer cell-dominant ascites cultures (SL1-SL4). (**C**) Module score heatmap from baseline RNA-seq of eight ascites-derived cultures. Values show batch-corrected module z-scores. Cultures were grouped by the H&E morphology of the corresponding primary tumor. (**D**) Violin and box plots of selected module scores in stroma-dominant and cancer cell-dominant groups. Each point represents one ascites-derived culture. Cohen’s d and q values are shown for each module. (**E**) Focused real-time PCR analysis of representative MAPK/TGF-beta pathway genes. Expression values were normalized to ACTB. Error bars indicate technical variation. This assay was performed as independent focused support rather than cohort-wide validation. (**F**) Representative H&E and IHC images from M-type and D-type tumors. CD44v6 staining was evaluated in tumor epithelial regions of primary tumors and metastatic lymph node lesions. VIM staining was evaluated in the stromal compartment. (**G**) Quantification of CD44v6 epithelial H-like scores and representative VIM stromal DAB-positive area. Each CD44v6 point is a case-level mean from multiple tumor epithelial ROIs. Group differences are reported descriptively because of the small number of independent cases. (**H**) Descriptive quantification of VIM stromal H-like scores from representative stromal ROIs. This panel was used to illustrate stromal/mesenchymal compartment enrichment rather than to establish cohort-level statistical inference. In the heatmaps, warm red colors indicate higher relative module or gene abundance, cool blue colors indicate lower abundance, and pale colors indicate intermediate values.

We analyzed baseline RNA-seq from eight independent patient-derived malignant ascites cultures. Clinicopathological and culture characteristics of the eight ascites-derived culture cases are summarized in Table 2, comprising four cultures derived from M-type primary tumors and four derived from D-type primary tumors. We next asked whether the morphology-associated cancer cell programs identified in the primary tumor were still detectable after dissemination. The corresponding primary tumors were classified by H&E morphology as stroma-dominant or differentiated cancer cell-dominant (Figure 6A,B).

**Table 2:** Clinicopathological and culture characteristics of the eight ascites-derived culture cases.

	Stroma-Dominant				Differentiated Cancer-Cell-Dominant			
Characteristic	SH1	SH2	SH3	SH4	SL1	SL2	SL3	SL4
**Timing of ascites collection**	At surgery	Before treatment	At surgery	At surgery	At ascites accumulation	At surgery	At ascites accumulation	At surgery
**Passage number at RNA extraction**	7	6	6	5	3	7	4	4
**Culture duration before RNA extraction**	1 month	1 month	1 month	1 month	1 month	2 months	2.5 month	1 month
**Prior systemic treatment**	None	None	None	None	ICI	None	FOLFOX/FOLFIRI	None
**Primary tumor location**	A	R	R	R	A	T	R	T
**Stage**	IV	IV	IV	IV	IV	IV	IV	IV
**Metastatic site(s)**	P	HEP	P	HEP	LN	P	P, PUL	P
**Original ascites cytology or cell block available**	No	No	No	No	No	No	No	No
**Formal epithelial/stromal purity assessment**	Not performed	Not performed	Not performed	Not performed	Not performed	Not performed	Not performed	Not performed
**MSI status**	MSI-H	Non-MSI-H	Non-MSI-H	Non-MSI-H	MSI-H	Non-MSI-H	Non-MSI-H	MSI-H
**RAS/BRAF status**	Not available	RAS/BRAF wild type	RAS/BRAF wild type	RAS/BRAF wild type	Not available	RAS/BRAF wild type	KRAS-mutant	Not available

Ascites-derived cultures were established by adherent propagation under uniform culture conditions. No FACS-based epithelial cell purification was performed. A, ascending colon; HEP, hepatic metastasis; ICI, immune checkpoint inhibitor; LN, lymph node metastasis; MSI-H, microsatellite instability-high; P, peritoneal metastasis; PUL, pulmonary metastasis; R, rectum; T, transverse colon. MSI-H, microsatellite instability-high; Non-MSI-H, tumors without MSI-H; RAS/BRAF wild type, no detected pathogenic mutation in the tested RAS and BRAF genes.

Cultures derived from stroma-dominant tumors showed higher core adaptive composite, ECM/integrin, EMT/pEMT, YAP/TAZ-TEAD, DTP/persister, and invasive epithelial scores (Figure 6C,D). ECM/integrin gave the largest separation (Cohen’s d = 7.07), followed by the core adaptive composite (d = 4.16), YAP/TAZ-TEAD (d = 3.03), DTP/persister (d = 2.93), and EMT/pEMT (d = 2.13). Stem/WNT and cell cycle/proliferation scores were unchanged. The culture phenotype was therefore concentrated in matrix engagement, mechanotransduction, and survival programs.

To assess whether canonical stromal/CAF gene content accounted for these differences, we repeated module scoring after excluding the same canonical stromal/CAF-associated genes used in the HEST sensitivity analysis. The group differences remained for the core adaptive composite (d = 3.73, q = 0.046), ECM/integrin (d = 4.79, q = 0.046), YAP/TAZ-TEAD (d = 3.03, q = 0.046), DTP/persister (d = 2.93, q = 0.046), and EMT/pEMT (d = 1.85, q = 0.046) (Supplementary Figure S9A,B). The culture-level separation therefore did not depend solely on canonical stromal genes included in the module definitions.

The original and stromal/CAF gene-excluded effect sizes are shown together in Supplementary Figure S9C.

Focused real-time PCR provided an independent comparison of selected MAPK/TGF-beta pathway genes. KRAS, MAP2K1, MAPK1, SMAD3, and TGFBR1 were higher in M-type-derived than D-type-derived ascites cells after normalization to ACTB; SMAD2 showed the same direction with greater variability (Figure 6E) (Supplementary Table S10). The assay reproduced the pathway direction observed in the transcriptomic analysis.

IHC extended the comparison to primary tumors and metastatic lymph node lesions. CD44v6 staining in tumor epithelial regions was higher in M-type than D-type primary tumors (mean H-like score 50.2 versus 4.3; Cohen’s d = 4.94; exact *p* = 0.10), and the relative difference persisted in metastatic lymph node lesions (47.0 versus 3.5; d = 2.80) (Figure 6F,G). VIM staining was concentrated in the broader stromal compartment of the representative M-type tumor (Figure 6F,H). The protein-level findings paralleled the morphology-associated RNA programs.

Matrix-engagement, mechanotransduction, and persister-like programs remained associated with stroma-dominant primary tumor morphology after dissemination and ex vivo expansion. Real-time PCR and IHC detected concordant morphology-associated features at RNA and protein levels.

### 3.7. Putative Cancer–CAF Interaction Analysis Nominates Reciprocal Stromal Signaling Programs

We also scored putative cancer–CAF interaction axes to determine whether stroma-dominant cultures carried coordinated candidate signaling programs. The analysis evaluated expression-inferred CAF-to-cancer and cancer-to-CAF axes in stroma-dominant and cancer cell-dominant cultures.

**Figure 7:**
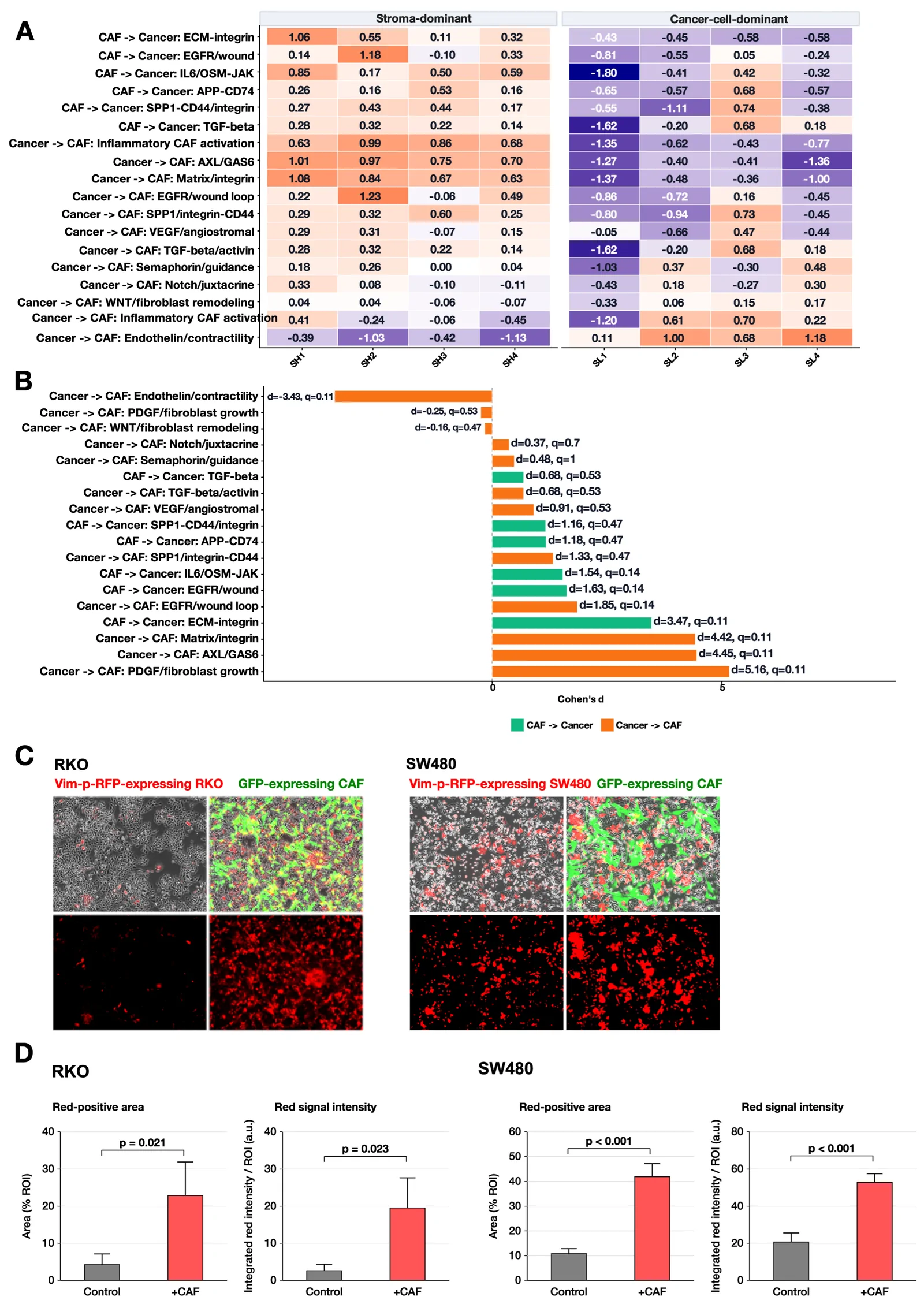
Putative cancer–CAF communication analysis and CAF co-culture support a stromal EMT-like niche. (**A**) Heatmap of putative directional pair-axis z-scores for expression-inferred CAF-to-cancer and cancer-to-CAF signaling programs in stroma-dominant and cancer cell-dominant ascites-derived cultures. (**B**) Effect-size ranking of putative directional cancer–CAF pair-axis scores. Green bars indicate CAF-to-cancer axes and orange bars indicate cancer-to-CAF axes; positive Cohen’s d indicates enrichment in stroma-dominant cultures. (**C**) Representative images of Vim-p-RFP-expressing RKO and SW480 colorectal cancer cells cultured alone or co-cultured with GFP-expressing CAFs. Upper panels show merged phase-contrast and fluorescence images, and lower panels show the RFP reporter channel. CAF co-culture increased vimentin promoter-driven RFP reporter activity in both RKO and SW480 cells. (**D**) Quantification of Vim-p-RFP reporter activity in colorectal cancer cells cultured with or without GFP-expressing CAFs. In the heatmaps, warm red colors indicate higher relative module or gene abundance, cool blue colors indicate lower abundance, and pale colors indicate intermediate values.

Stroma-dominant cultures showed broad enrichment of putative CAF-to-cancer signaling axes, including ECM-integrin, EGFR/wound, IL6/OSM-JAK, SPP1-CD44/integrin, and TGF-beta-related signaling (Figure 7A). In parallel, several putative cancer-to-CAF axes were also elevated in stroma-dominant cultures, including inflammatory CAF activation, AXL/GAS6, matrix/integrin, EGFR/wound loop, SPP1/integrin-CD44, and VEGF/angiostromal signaling (Figure 7A).

The largest positive differences involved cancer-to-CAF inflammatory CAF activation (Cohen’s d = 5.16, q = 0.11), cancer-to-CAF AXL/GAS6 (d = 4.45, q = 0.11), cancer-to-CAF matrix/integrin (d = 4.42, q = 0.11), and CAF-to-cancer ECM-integrin (d = 3.47, q = 0.11) (Figure 7B). IL6/OSM-JAK, EGFR/wound, and EGFR/wound loop axes showed moderate effects (d = 1.54–1.85, q = 0.14). These pair-axis comparisons were exploratory and did not meet the 5% false-discovery-rate threshold.

### 3.8. CAF Co-Culture Increases EMT Reporter Activity in Colorectal Cancer Cells

We tested whether CAFs could increase EMT-like transcriptional activity using colorectal cancer cells carrying a vimentin promoter-driven RFP reporter [27]. RKO and SW480 reporter cells were cultured alone or with GFP-expressing CAFs. CAF co-culture increased both the number and intensity of RFP-positive cancer cells in each cell line (Figure 7C).

Quantification confirmed this effect: both the red-positive area and the integrated red signal intensity per ROI were significantly higher in CAF co-culture than in monoculture for RKO (*p* = 0.021 and *p* = 0.023, respectively) and SW480 (both *p* < 0.001) cells (Figure 7D).

## 4. Discussion

Stroma-dominant CRC differed from gland-forming CRC in both the stromal and cancer cell compartments. M-type tissue contained an expanded matrix-remodeling CAF compartment, while EPCAM/KRT-high cancer cell regions showed higher EMT/pEMT, ECM/integrin, YAP/TAZ, and DTP/persister activity. These programs were present in tumor core regions and became strongest at CAF-facing interfaces. Mesenchymal CRC is therefore characterized by a spatially organized cancer–stroma niche, not simply by an increased amount of stroma.

Within M-type cancer cell regions, the four programs marked connected aspects of the same phenotype. ECM/integrin reflected contact with the surrounding matrix, YAP/TAZ reflected mechanical signaling, and DTP/persister genes reflected survival under stress. Their co-occurrence distinguished M-type cancer cells from the differentiated, gland-forming compartment of D-type tumors. Their increase along the CAF neighbor gradient placed the strongest expression at the tumor–stroma interface.

The two compartments carried complementary signaling programs. CAF/stromal cells expressed matrix, SPP1, TGF-beta, and inflammatory factors near cancer cells with high activity of the four primary programs. The cancer cell compartment expressed TGF-beta/activin, PDGF/FGF, Notch/WNT, and angiostromal-remodeling ligands. In public datasets and the VIM promoter experiment, CAF exposure increased EMT- and matrix-related activity in CRC cells. These results support a reciprocal niche in which stromal remodeling and cancer cell adaptation are linked.

The spatial pattern also suggests how M-type architecture may develop. CAF-rich stroma can reinforce pEMT and survival programs in adjacent cancer cells, while cancer cells expressing stromal recruitment ligands can expand or remodel the CAF compartment. The two directions may reinforce each other and stabilize the desmoplastic architecture.

Evidence for a cancer cell component came from three sources. The four programs remained elevated in EPCAM/KRT-high tumor core regions with low stromal content. In two single-cell datasets, samples containing more cancer cells with this four-program state also contained larger CAF fractions. The same program pattern was detected in ascites-derived cultures after removal from the primary tissue. Spatial, single-cell, and culture data therefore converged on a cancer cell component within the M-type phenotype.

Excluding canonical stromal/CAF genes from the module definitions left the principal effects intact in HEST EPCAM/KRT-high spots, epithelial-dense tumor core spots, and ascites-derived cultures. The ECM/integrin effect decreased after removal of collagen, fibronectin, and related stromal genes but remained significant. Modules without excluded genes were essentially unchanged. This analysis strengthens the epithelial-enriched interpretation without treating spatial spots or bulk cultures as purified malignant cell populations.

The ascites cultures extended this association beyond the primary tumor. After 1–2.5 months of ex vivo growth, cultures from stroma-dominant primaries still showed higher ECM/integrin, YAP/TAZ, persister, and stress tolerance programs and lower intestinal differentiation. The molecular phenotype of the disseminated cultures therefore remained linked to the stromal morphology of the corresponding primary tumor.

The clinical cohorts linked the same morphology and programs to outcome. M-pattern morphology predicted overall survival in TCGA after adjustment for age, stage, site, MSI status, and tumor purity. The individual modules added no further information once morphology entered the TCGA models, consistent with the broad biological content of the histological pattern. In GSE39582, EMT/pEMT, ECM/integrin, DTP/persister, invasive epithelial activity, and the composite niche score predicted recurrence after adjustment for stage, location, MMR status, and adjuvant chemotherapy. YAP/TAZ-TEAD separated the spatial groups but did not predict recurrence, indicating that spatial enrichment and prognosis measured related but different features of the niche.

IHC and focused real-time PCR reproduced selected features outside the RNA-seq cohorts. CD44v6 staining was higher in M-type tumor epithelium and retained the same relative pattern in metastatic lymph nodes. VIM marked the broader stromal compartment of M-type tumors. In an independent ascites cell pair, real-time PCR showed higher MAPK/TGF-beta pathway expression in the M-type-derived sample. These experiments connected the transcriptomic pattern to protein expression and targeted gene measurements.

This study has several limitations. The spatial analysis comprised 20 HEST-1K sections summarized into 12 patient/tissue units, and spatial spots may contain transcripts from more than one cell type. Ambient RNA and doublets may also affect the single-cell datasets. The IHC and focused real-time PCR comparisons involved small independent series. The ascites profiles came from bulk cultures without EPCAM sorting, serial passage sampling, or matched uncultured ascites. These data cannot distinguish persistence of a cancer cell state from selection during dissemination or culture, and the ligand–receptor scores measure coexpression rather than direct signaling. The mechanisms that establish and maintain the M-type phenotype therefore remain unresolved. The ascites culture comparison was limited to eight independent patient-derived cultures, with four cultures in each morphology group. Its effect estimates may therefore be influenced by individual cases, and this analysis should be interpreted as exploratory support rather than as standalone cohort-level validation.

Future studies should test the reproducibility and clinical relevance of the M-type and D-type phenotypes in larger independent cohorts. A fundamental unanswered question is how these contrasting tissue architectures arise. It remains unclear whether M-type and D-type tumors are specified as distinct biological states early in tumorigenesis, whether one phenotype emerges from the other during tumor progression, or whether reversible transitions are triggered by CAF interactions, treatment, or other microenvironmental pressures. Patient-derived colorectal cancer organoids cultured with matched CAFs could recreate the spatial relationships identified here and allow direct perturbation of candidate pathways. Such models may determine whether CAFs shape the cancer cell state, cancer cells establish the CAF-rich compartment, or reciprocal signaling initiates and maintains a transition toward the M-type phenotype.

## 5. Conclusions

Stroma-dominant mesenchymal CRC is not simply CRC with more stroma. It represents a spatially organized tumor ecosystem in which expanded CAF-rich compartments are associated with cancer cell EMT/plasticity, matrix engagement, YAP/TAZ mechanotransduction, and persister-like survival. Components of this cancer cell state remained detectable after dissemination in ascites-derived cultures and were independently supported by focused real-time PCR and IHC, including metastatic lymph node lesions. Together with their associations with recurrence, these findings indicate that the adverse biology of mesenchymal CRC involves both stromal architecture and a distinct, morphology-associated cancer cell state.

## Figures and Tables

**Table 1:** Multivariable Cox proportional hazards analyses in TCGA and GSE39582.

Model	Predictor	HR	95% CI	*p* Value	BH *q* Value
**A. TCGA overall survival: morphology models**					
Primary model ^a^	M-type versus D-type morphology	2.94	1.75–4.95	<0.001	–
CMS sensitivity ^b^	M-type versus D-type morphology	2.41	1.28–4.55	0.0066	–
**B. TCGA overall survival: module terms after inclusion of morphology**					
Joint model ^c^	Composite niche	1.05	0.82–1.35	0.683	0.933
Joint model	Core adaptive composite	1.10	0.69–1.75	0.692	0.933
Joint model	CAF/stroma component	1.06	0.80–1.39	0.700	0.933
Joint model	EMT/pEMT	1.08	0.74–1.59	0.689	0.933
Joint model	ECM/integrin	1.00	0.70–1.43	0.990	0.990
Joint model	YAP/TAZ-TEAD	1.05	0.65–1.70	0.846	0.967
Joint model	DTP/persister	1.25	0.78–2.00	0.359	0.933
Joint model	Invasive epithelial	0.86	0.59–1.25	0.425	0.933
**C. GSE39582 recurrence-free survival: continuous-score models**					
Adjusted model ^d^	Composite niche	1.31	1.11–1.55	0.0017	0.0023
Adjusted model	Core adaptive composite	1.80	1.29–2.53	0.00065	0.0012
Adjusted model	CAF/stroma component	1.31	1.08–1.59	0.0070	0.0080
Adjusted model	EMT/pEMT	1.59	1.23–2.06	0.00049	0.0012
Adjusted model	ECM/integrin	1.56	1.21–2.03	0.00073	0.0012
Adjusted model	YAP/TAZ-TEAD	1.35	0.93–1.96	0.119	0.119
Adjusted model	DTP/persister	1.83	1.30–2.58	0.00048	0.0012
Adjusted model	Invasive epithelial	1.78	1.35–2.36	<0.001	<0.001

^a^ The primary TCGA model included 426 patients and 77 deaths and was adjusted for age, stage, primary tumor location, MANTIS MSI status, and ABSOLUTE tumor purity. ^b^ The CMS sensitivity model additionally included CMS subtype (*n* = 275, 58 deaths). ^c^ Each TCGA joint model included H&E morphology, one continuous module score, age, stage, primary tumor location, MANTIS MSI status, and ABSOLUTE tumor purity. M-type morphology retained prognostic information, whereas the module terms did not provide additional overall survival information beyond morphology. ^d^ Each GSE39582 model included one continuous module score together with stage, tumor location, MMR status, and adjuvant chemotherapy (*n* = 492, 140 recurrence events). Module score hazard ratios represent a one-unit increase in the original mean-gene-z score. No second standardization was applied. BH, Benjamini–Hochberg; CI, confidence interval; HR, hazard ratio; MMR, mismatch repair status; MSI, microsatellite instability.

## Data Availability

TCGA-COAD and TCGA-READ RNA-seq, clinical, and pathology-image resources were obtained through the Genomic Data Commons, with overall-survival annotations from the TCGA Clinical Data Resource (last accessed 17 August 2026). HEST-1K spatial transcriptomic data were downloaded from MahmoodLab/hest on Hugging Face (version 1.3.0; accessed 27 June 2026); the analyzed CRC section IDs and patient/tissue-unit mapping are listed in Supplementary Table S1. GEO datasets GSE39582, GSE132465, GSE144735, GSE214569, and GSE198697 were last accessed on 17 August 2026. The institutional ascites-derived culture RNA-seq data do not currently have a public accession. De-identified data may be made available by the corresponding author on reasonable request, subject to institutional and ethics review requirements.

## Supplementary Materials

The following supporting information can be downloaded at: https://www.mdpi.com/article/10.3390/biom16091261/s1.

## Abbreviations

BH	Benjamini–Hochberg
CAF	cancer-associated fibroblast
CI	confidence interval
CMS	consensus molecular subtype
CRC	colorectal cancer
DAB	3,3′-diaminobenzidine
DTP	drug-tolerant persister
ECM	extracellular matrix
EMT	epithelial–mesenchymal transition
EPCAM	epithelial cell adhesion molecule
GEO	Gene Expression Omnibus
H&E	hematoxylin and eosin
HEST	Histology-based Spatial Transcriptomics
HR	hazard ratio
IHC	immunohistochemistry
KRT	keratin
MMR	mismatch repair
MSI	microsatellite instability
OS	overall survival
pEMT	partial epithelial–mesenchymal transition
qPCR	quantitative real-time PCR
RFS	recurrence-free survival
RNA-seq	RNA sequencing
scRNA-seq	single-cell RNA sequencing
TAZ	transcriptional coactivator with PDZ-binding motif
TCGA	The Cancer Genome Atlas
TEAD	TEA domain transcription factor
VIM	vimentin
YAP	Yes-associated protein
