# Supplementary material for: Stroma-Dominant Colorectal Cancer Harbors CAF-Rich Spatial Architecture and EMT-Associated Cancer Cell Plasticity Detectable After Dissemination
Source: Biomolecules. 2026 Aug 31;16(9):1261. doi: 10.3390/biom16091261 (PMC13604046; doi:10.3390/biom16091261)
Supplement: Supplementary file 1 [file biomolecules-16-01261-s001.zip › biomolecules-4482148-SM/biomolecules-4482148-SuppleFigsR1.pdf]

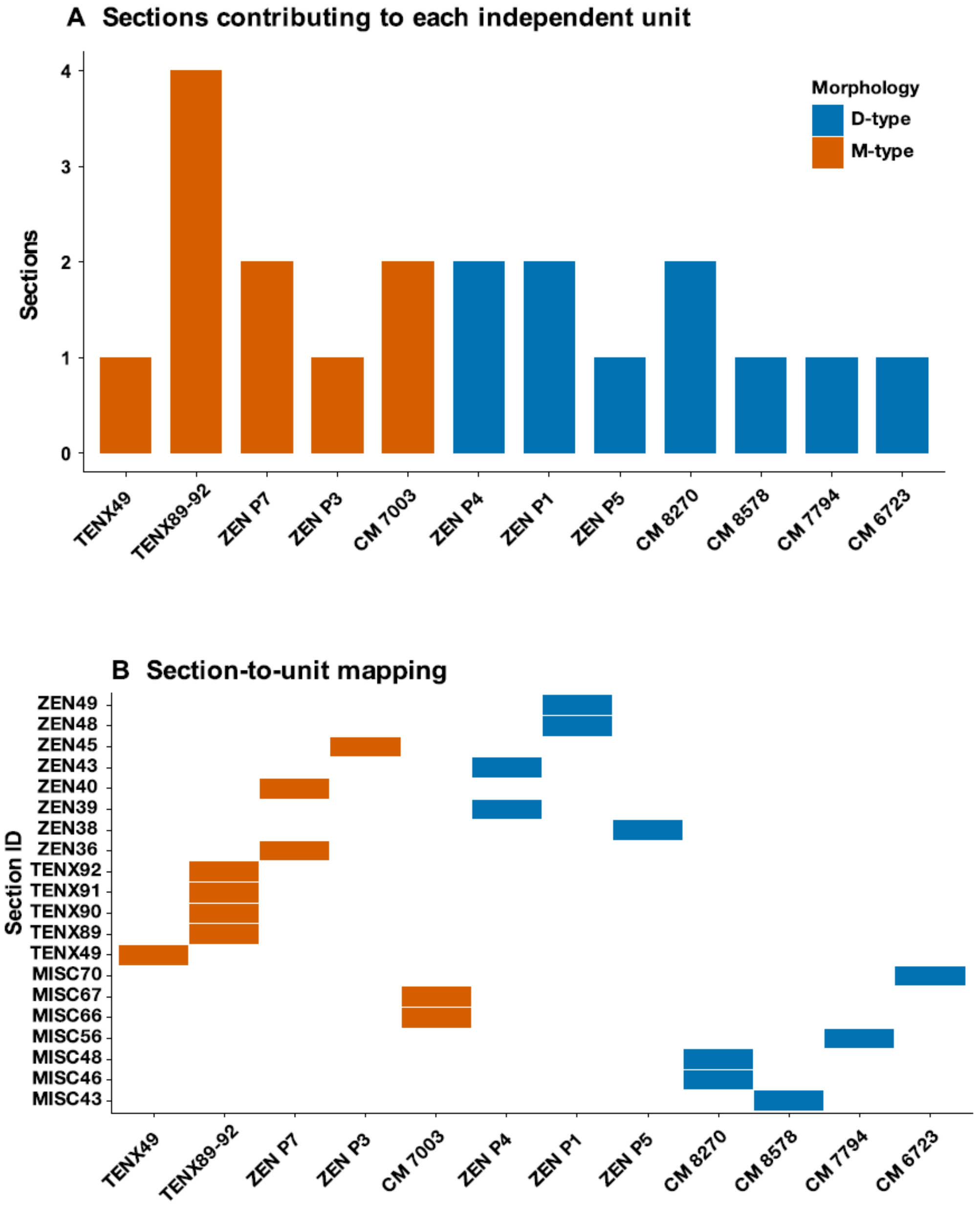

Supplementary Figure S1. Composition of the HEST-1K spatial cohort and mapping of sections to independent analysis units. A. Number of HEST-1K sections contributing to each independent patient/tissue unit, colored by H&E morphology (orange, M-type; blue, D-type). B. Mapping of the 20 analyzed sections to 12 independent patient/tissue units. Ten sections were assigned to five M-type units and ten sections to seven D-type units. Serial or otherwise related sections were summarized within the same unit. The patient/tissue unit, rather than the individual section or spatial spot, was used as the independent observation for between-morphology comparisons.

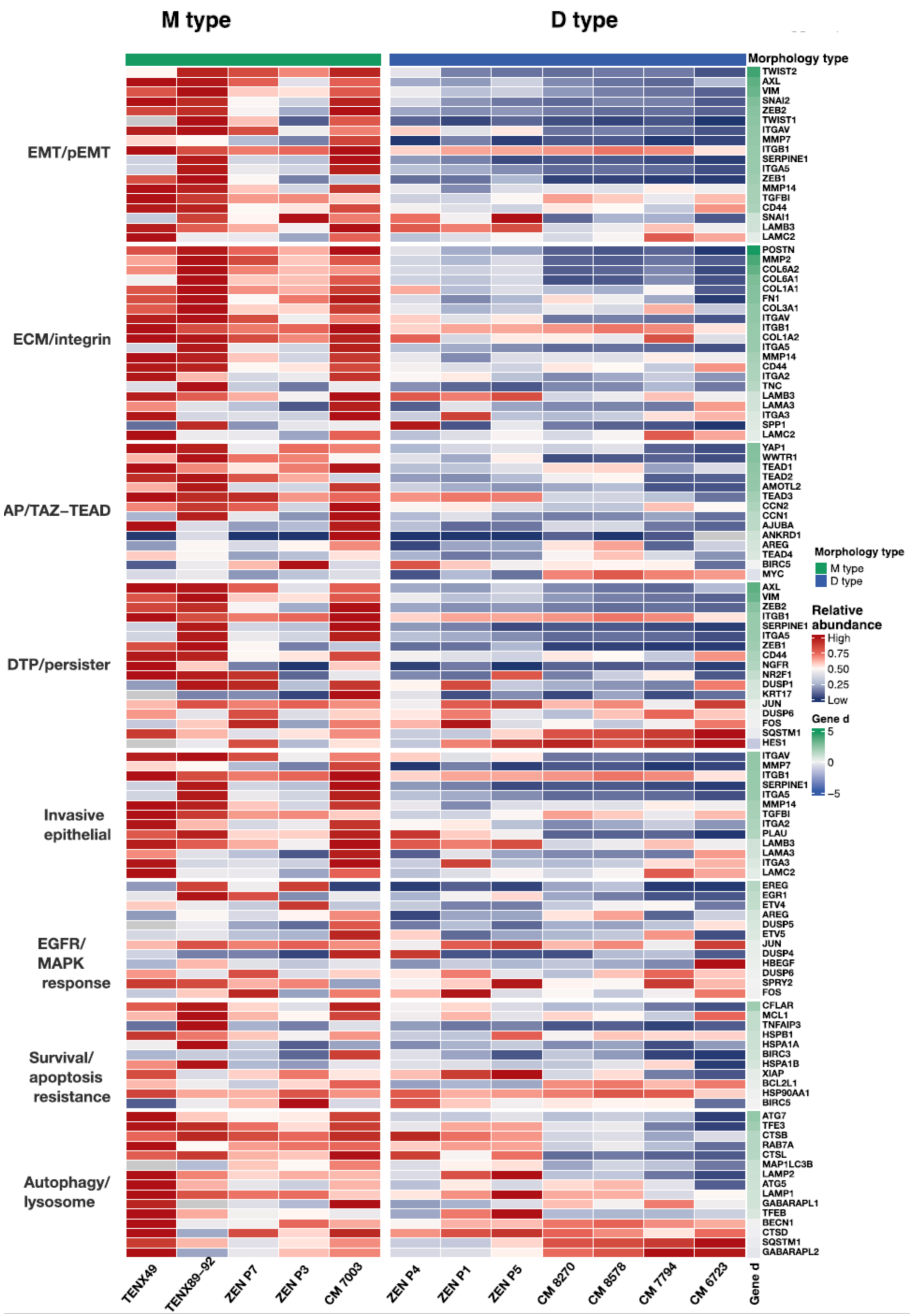

Supplementary Figure S2. Constituent-gene patterns of epithelial-enriched adaptive and stress-response modules. Gene-level heatmap of the prespecified EMT/pEMT, ECM/integrin, YAP/TAZ-TEAD, DTP/persister, invasive epithelial, EGFR/MAPK-response, survival/apoptosis-resistance, and autophagy/lysosome modules in EPCAM/KRT-high spots. Columns represent the 12 independent patient/tissue units, ordered as five M-type and seven D-type units (green and blue column headers, respectively). For each gene, log-normalized expression was divided by its 95th-

percentile reference derived from the 55-section CRC HEST panel and truncated to the interval from 0 to 1. Red indicates higher and blue indicates lower relative abundance. The side annotation labeled Gene d shows the unit-level Cohen's d for M-type versus D-type; green indicates positive values and blue indicates negative values. Constituent genes were prespecified and were not selected according to the observed morphology-group difference.

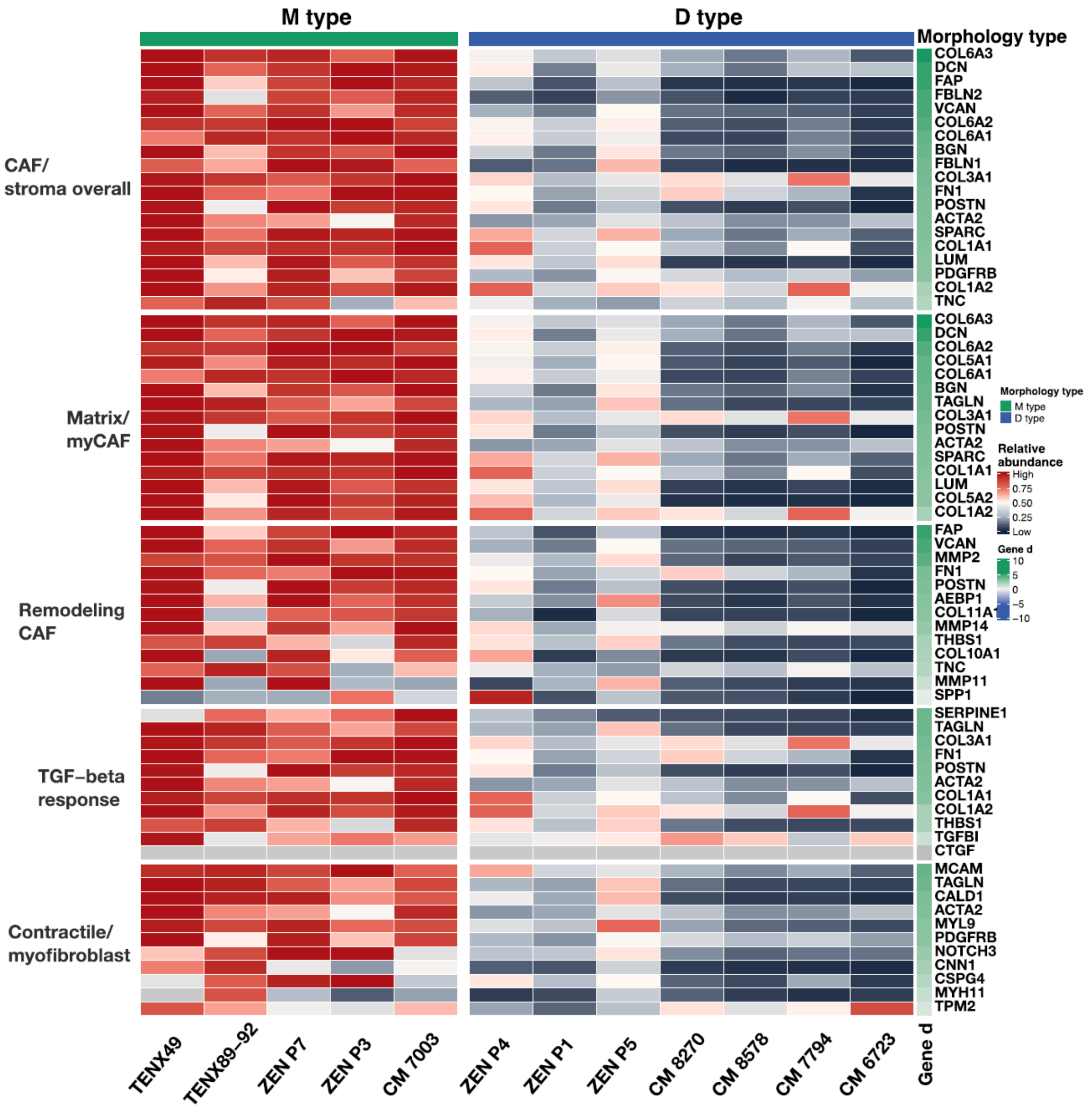

Supplementary Figure S3. Constituent-gene patterns of M-type-enriched CAF/stroma programs. Gene-level heatmap of the overall CAF/stroma, matrix/myCAF, remodeling-CAF, TGF-beta-response, and contractile/myofibroblast programs in non-epithelial spatial spots. Columns represent five M-type and seven D-type independent

patient/tissue units (green and blue column headers, respectively). Relative abundance was calculated from the 55-section CRC HEST 95th-percentile reference and bounded from 0 to 1; red indicates higher and blue indicates lower abundance. The Gene d annotation shows the unit-level Cohen's d for M-type versus D-type, with green indicating positive values and blue indicating negative values. These compartment-level programs summarize stromal composition and state and do not identify a single purified fibroblast population.

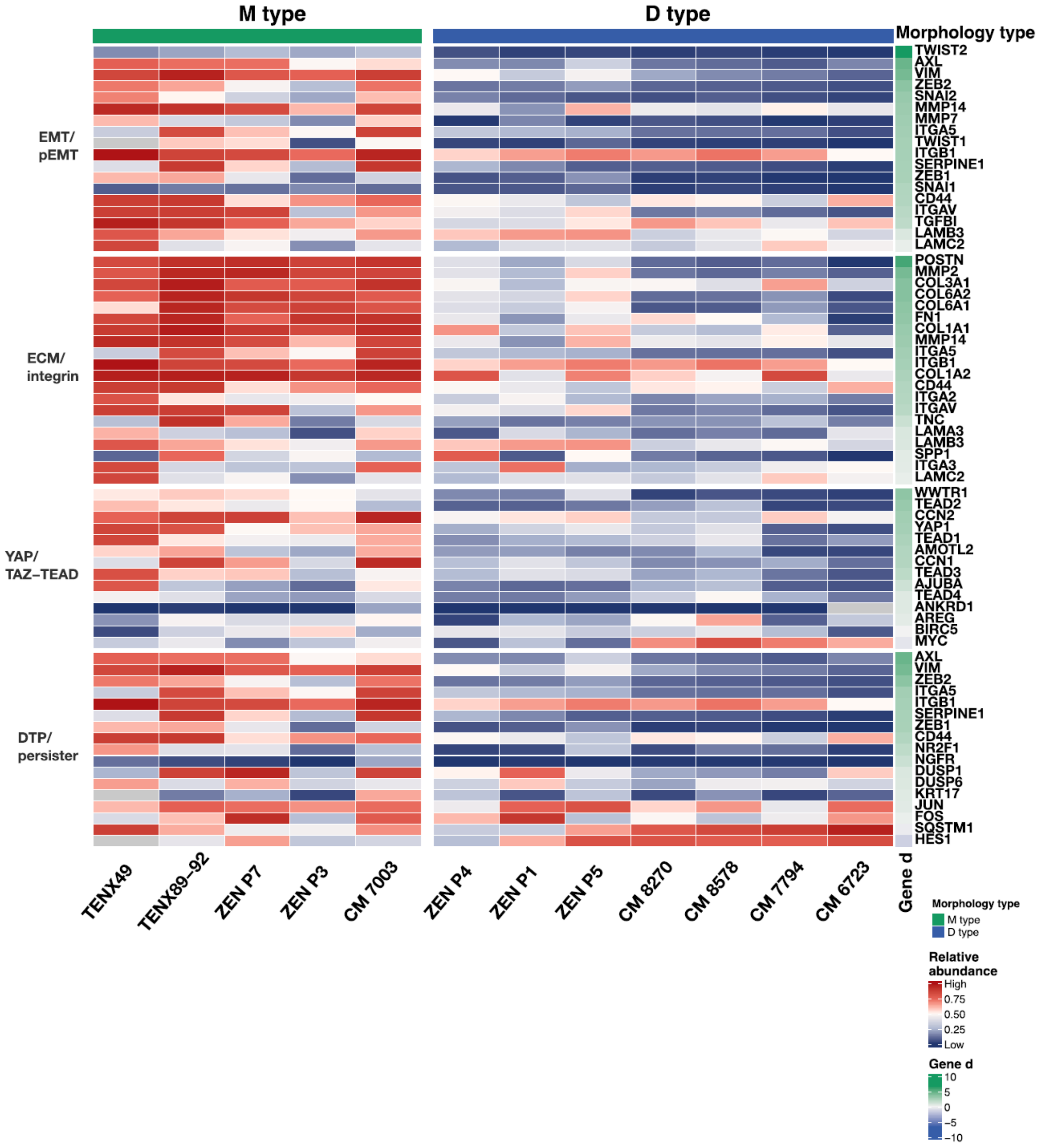

Supplementary Figure S4. Constituent-gene patterns of adaptive modules in CAF-

neighbor epithelial spots. Gene-level heatmap of EMT/pEMT, ECM/integrin, YAP/TAZ-TEAD, and DTP/persister programs in EPCAM/KRT-high spots located in CAF-rich neighborhoods. Columns represent five M-type and seven D-type independent patient/tissue units (green and blue column headers, respectively). Values are unit-level summaries of gene relative abundance calculated against the fixed 55-section CRC HEST 95th-percentile reference and bounded from 0 to 1. Red indicates higher and blue indicates lower abundance. The Gene d annotation shows the unit-level Cohen's d for M-type versus D-type, with green indicating positive values and blue indicating negative values. Because spatial spots can contain transcripts from more than one cell type, the heatmap represents cancer-cell-enriched tumor-stroma interface regions rather than purified malignant cells.

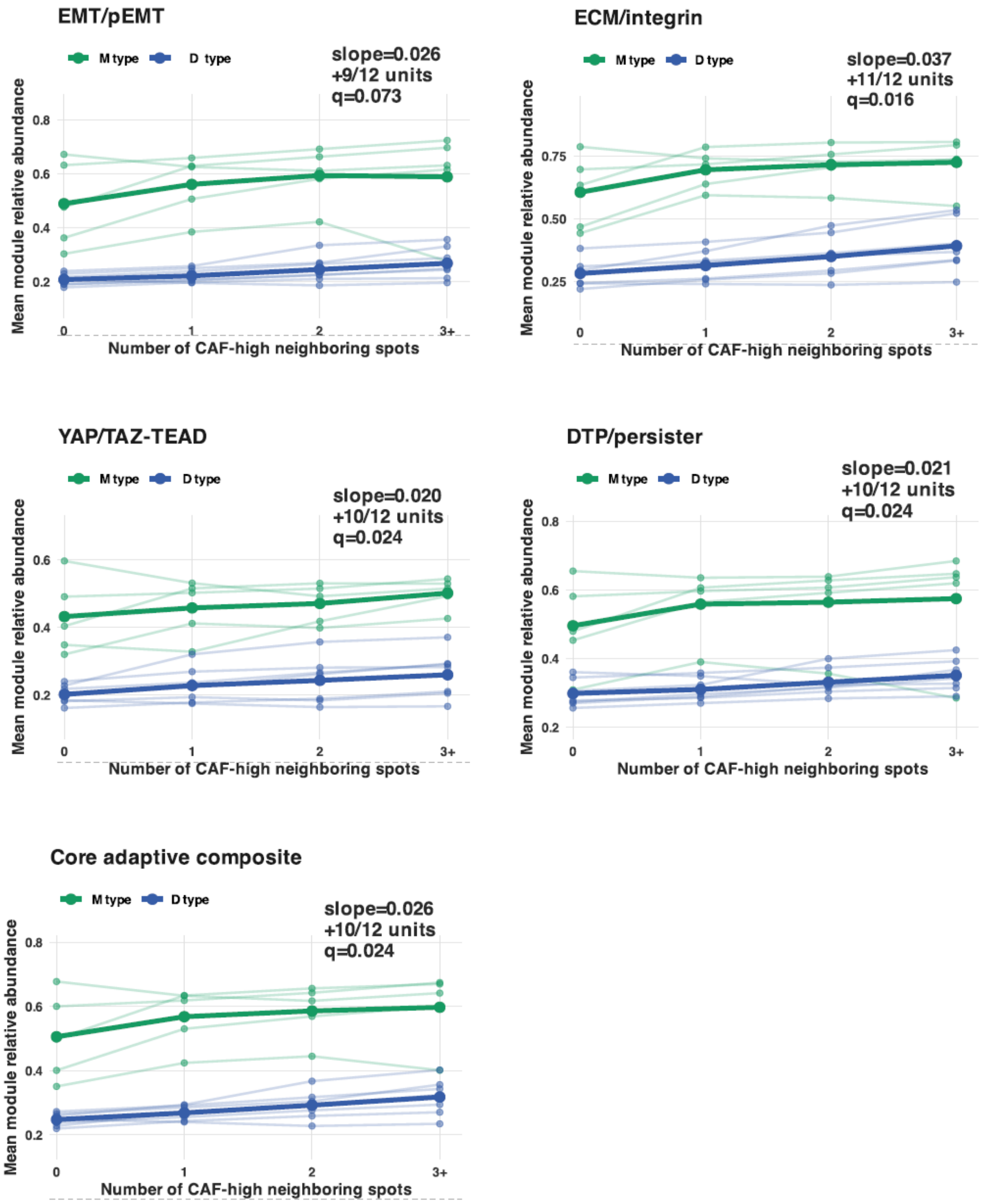

Supplementary Figure S5. Epithelial adaptive-program abundance across the CAF-neighbor gradient. EPCAM/KRT-high spots were grouped by the number of immediate CAF/stroma-high hexagonal neighbors within each section: 0, 1, 2, or 3 or more. Panels show EMT/pEMT, ECM/integrin, YAP/TAZ-TEAD, DTP/persister, and the core adaptive composite. Thin lines represent independent patient/tissue units and thick lines represent morphology-group means; M-type is green and D-type is blue. The y-axis shows mean module relative abundance calculated from the fixed 55-section CRC HEST reference. The displayed slope summarizes the change across CAF-neighbor categories, and +n/12

units gives the number of independent units with a positive slope. q values were adjusted by the Benjamini-Hochberg method within the prespecified CAF-neighborhood analysis family.

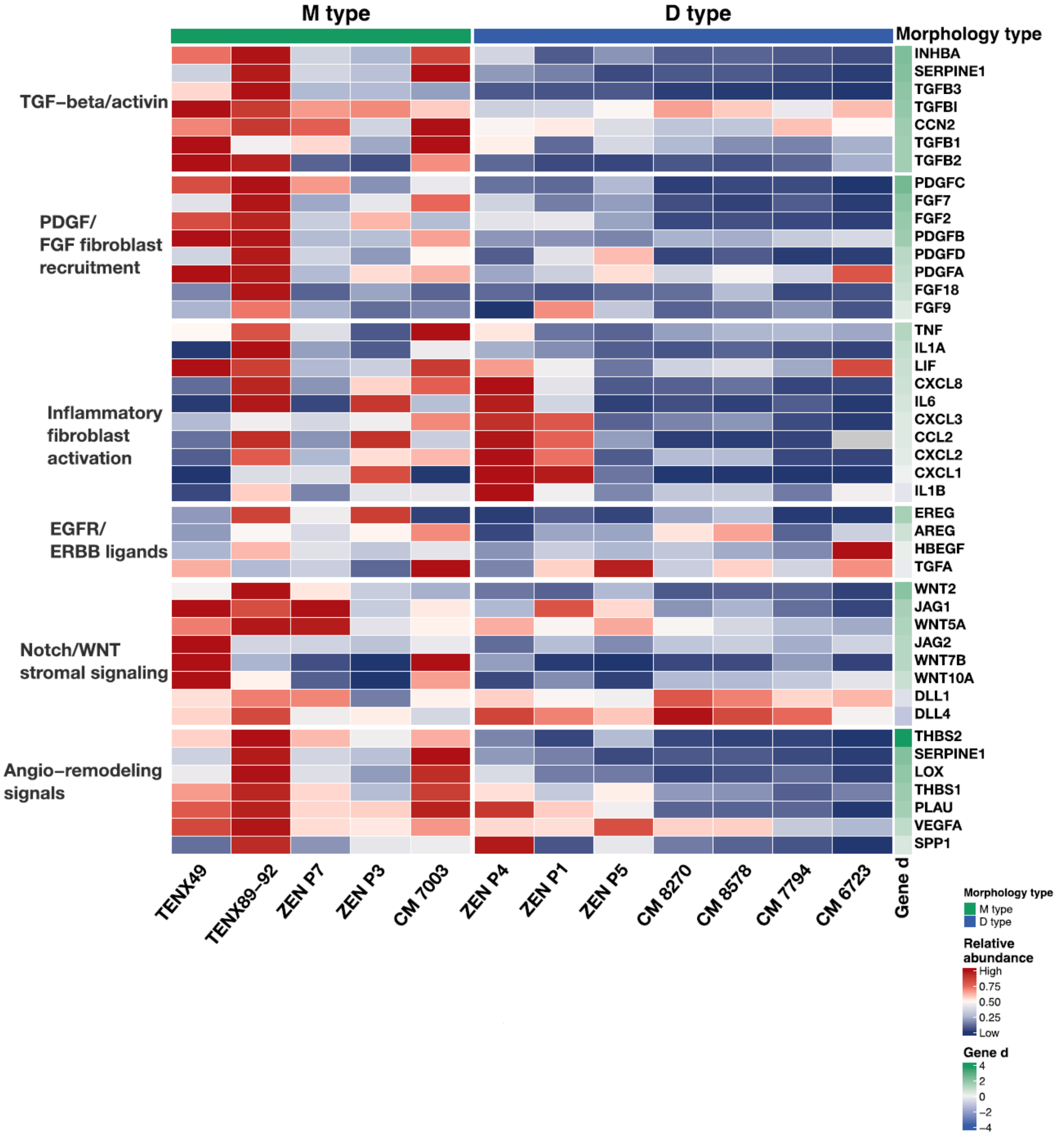

Supplementary Figure S6. Constituent cancer-cell ligand programs associated with CAF/stroma recruitment. Gene-level heatmap of prespecified cancer-cell ligand programs in EPCAM/KRT-high spots, organized into TGF-beta/activin, PDGF/FGF fibroblast recruitment, inflammatory fibroblast activation, EGFR/ERBB ligands, Notch/WNT stromal signaling, and angio-remodeling axes. Columns represent five M-type and seven D-type independent patient/tissue units (green and blue column headers,

respectively). Relative abundance was calculated against the fixed 55-section CRC HEST 95th-percentile reference and bounded from 0 to 1; red indicates higher and blue indicates lower abundance. The Gene d annotation shows the unit-level Cohen's d for M-type versus D-type, with green indicating positive values and blue indicating negative values. These directional labels denote expression-inferred candidate ligand programs in cancer-cell-enriched regions and do not establish ligand secretion, receptor activation, direct cell-cell communication, or causality.

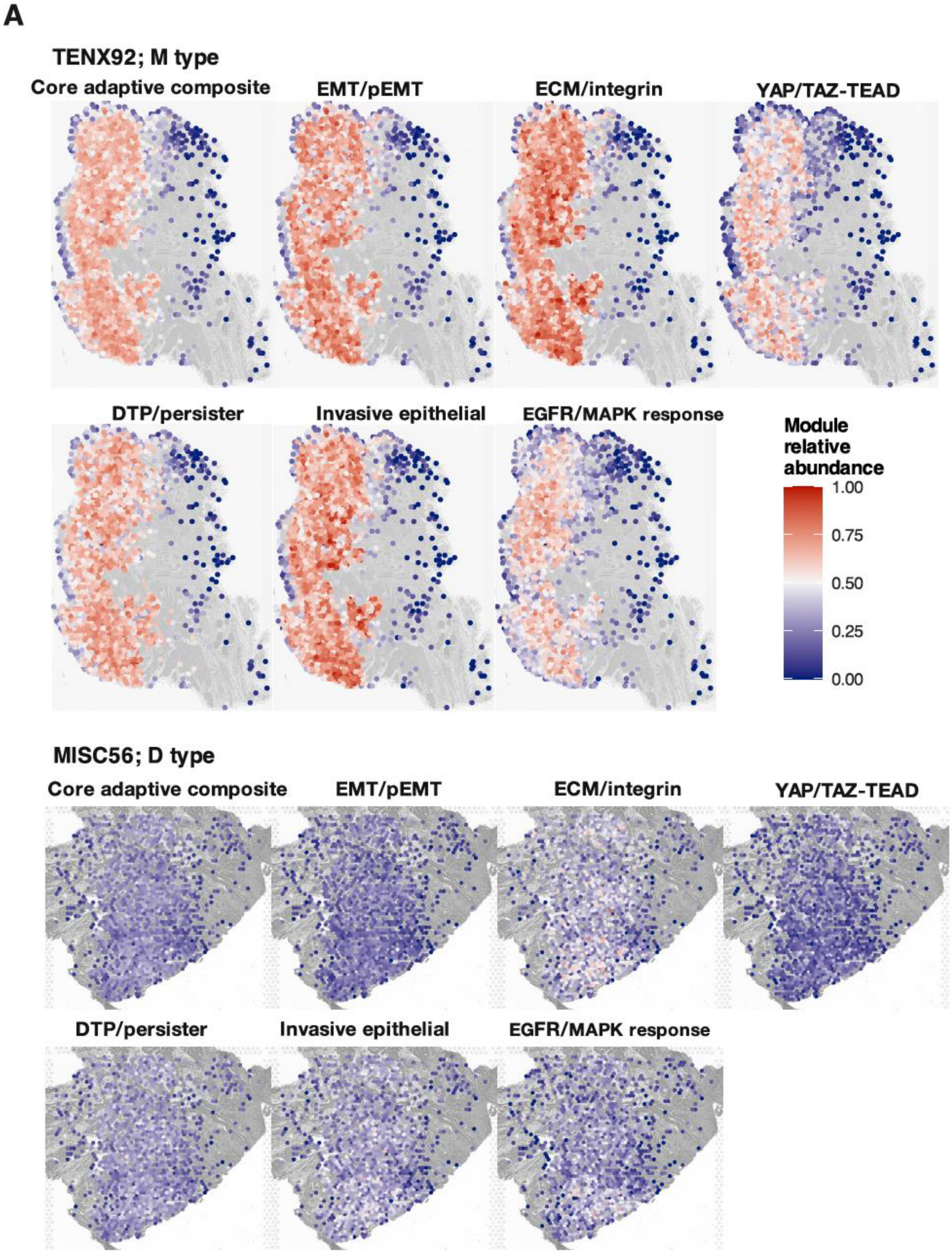
A
TENX92; M type
Core adaptive composite
EMT/pEMT
ECM/integrin
YAP/TAZ-TEAD
DTP/persister
Invasive epithelial
EGFR/MAPK response
Module relative abundance
1.00
0.75
0.50
0.25
0.00
MISC56; D type
Core adaptive composite
EMT/pEMT
ECM/integrin
YAP/TAZ-TEAD
DTP/persister
Invasive epithelial
EGFR/MAPK response

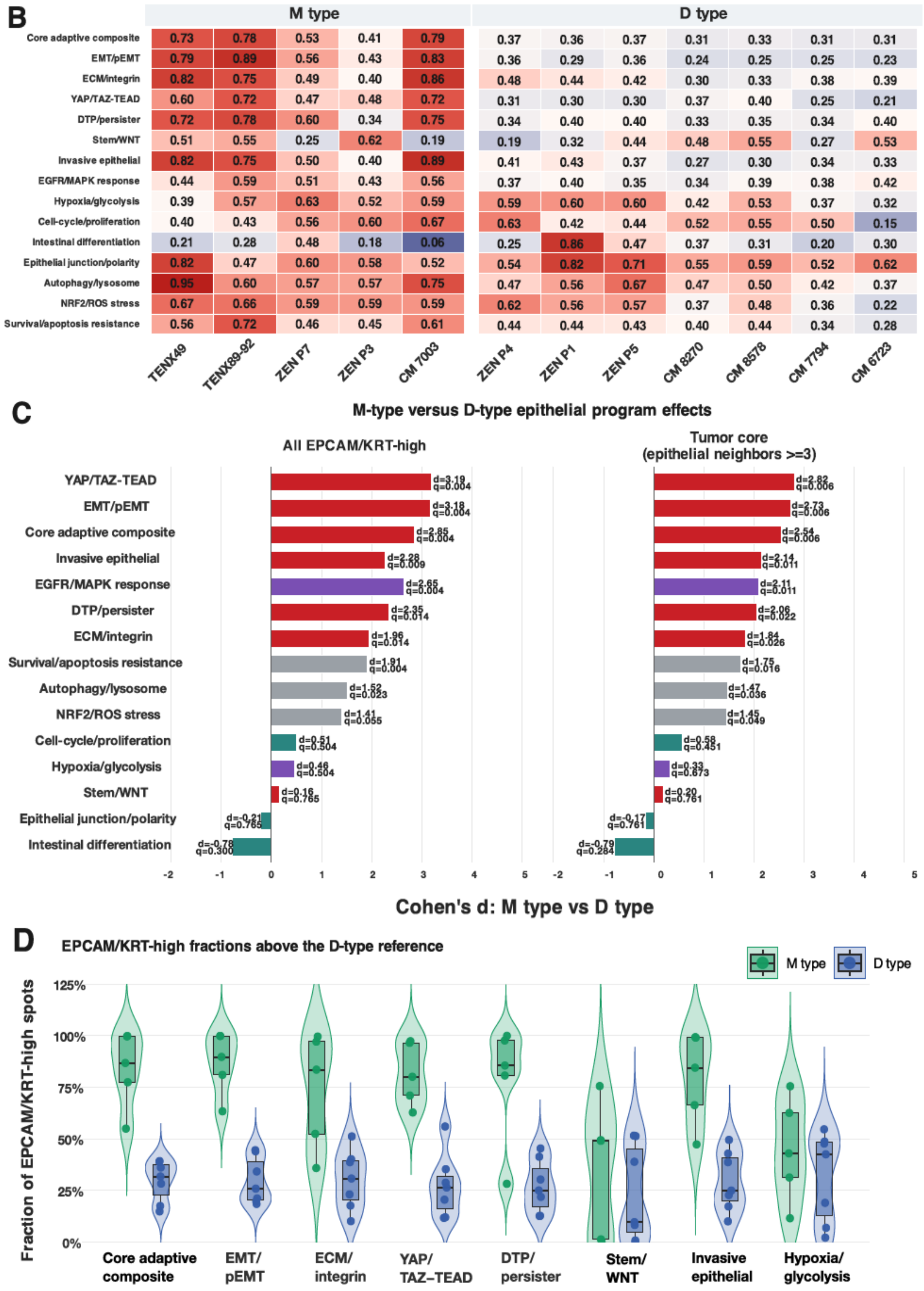
B
M type
D type
Core adaptive composite 0.73 0.78 0.53 0.41 0.79 0.37 0.36 0.37 0.31 0.33 0.31 0.31
EMT/pEMT 0.79 0.89 0.56 0.43 0.83 0.36 0.29 0.36 0.24 0.25 0.25 0.23
ECM/integrin 0.82 0.75 0.49 0.40 0.86 0.48 0.44 0.42 0.30 0.33 0.38 0.39
YAP/TAZ-TEAD 0.60 0.72 0.47 0.48 0.72 0.31 0.30 0.30 0.37 0.40 0.25 0.21
DTP/persister 0.72 0.78 0.60 0.34 0.75 0.34 0.40 0.40 0.33 0.35 0.34 0.40
Stem/WNT 0.51 0.55 0.25 0.62 0.19 0.19 0.32 0.44 0.48 0.55 0.27 0.53
Invasive epithelial 0.82 0.75 0.50 0.40 0.89 0.41 0.43 0.37 0.27 0.30 0.34 0.33
EGFR/MAPK response 0.44 0.59 0.51 0.43 0.56 0.37 0.40 0.35 0.34 0.39 0.38 0.42
Hypoxia/glycolysis 0.39 0.57 0.63 0.52 0.59 0.59 0.60 0.60 0.42 0.53 0.37 0.32
Cell-cycle/proliferation 0.40 0.43 0.56 0.60 0.67 0.63 0.42 0.44 0.52 0.55 0.50 0.15
Intestinal differentiation 0.21 0.28 0.48 0.18 0.06 0.25 0.86 0.47 0.37 0.31 0.20 0.30
Epithelial junction/polarity 0.82 0.47 0.60 0.58 0.52 0.54 0.82 0.71 0.55 0.59 0.52 0.62
Autophagy/lysosome 0.95 0.60 0.57 0.57 0.75 0.47 0.56 0.67 0.47 0.50 0.42 0.37
NRF2/ROS stress 0.67 0.66 0.59 0.59 0.59 0.62 0.56 0.57 0.37 0.48 0.36 0.22
Survival/apoptosis resistance 0.56 0.72 0.46 0.45 0.61 0.44 0.44 0.43 0.40 0.44 0.34 0.28
TENX49 TENX89-92 ZEN P7 ZEN P3 CM 7003 ZEN P4 ZEN P1 ZEN P5 CM 8270 CM 8578 CM 7794 CM 6723
C
M-type versus D-type epithelial program effects
All EPCAM/KRT-high
Tumor core (epithelial neighbors >=3)
YAP/TAZ-TEAD d=3.19 q=0.004 d=2.82 q=0.006
EMT/pEMT d=3.18 q=0.004 d=2.73 q=0.006
Core adaptive composite d=2.85 q=0.004 d=2.54 q=0.006
Invasive epithelial d=2.28 q=0.009 d=2.14 q=0.011
EGFR/MAPK response d=2.65 q=0.004 d=2.11 q=0.011
DTP/persister d=2.35 q=0.014 d=2.06 q=0.022
ECM/integrin d=1.96 q=0.014 d=1.84 q=0.026
Survival/apoptosis resistance d=1.91 q=0.004 d=1.75 q=0.016
Autophagy/lysosome d=1.52 q=0.023 d=1.47 q=0.036
NRF2/ROS stress d=1.41 q=0.055 d=1.45 q=0.049
Cell-cycle/proliferation d=0.51 q=0.504 d=0.58 q=0.451
Hypoxia/glycolysis d=0.46 q=0.504 d=0.33 q=0.673
Stem/WNT d=0.16 q=0.765 d=0.20 q=0.761
Epithelial junction/polarity d=-0.21 q=0.765 d=-0.17 q=0.761
Intestinal differentiation d=-0.78 q=0.300 d=-0.79 q=0.284
-2 -1 0 1 2 3 4 5 -2 -1 0 1 2 3 4 5
Cohen's d: M type vs D type
D
EPCAM/KRT-high fractions above the D-type reference
M type
D type
Fraction of EPCAM/KRT-high spots
125%
100%
75%
50%
25%
0%
Core adaptive composite
EMT/ pEMT
ECM/ integrin
YAP/ TAZ–TEAD
DTP/ persister
Stem/ WNT
Invasive epithelial
Hypoxia/ glycolysis

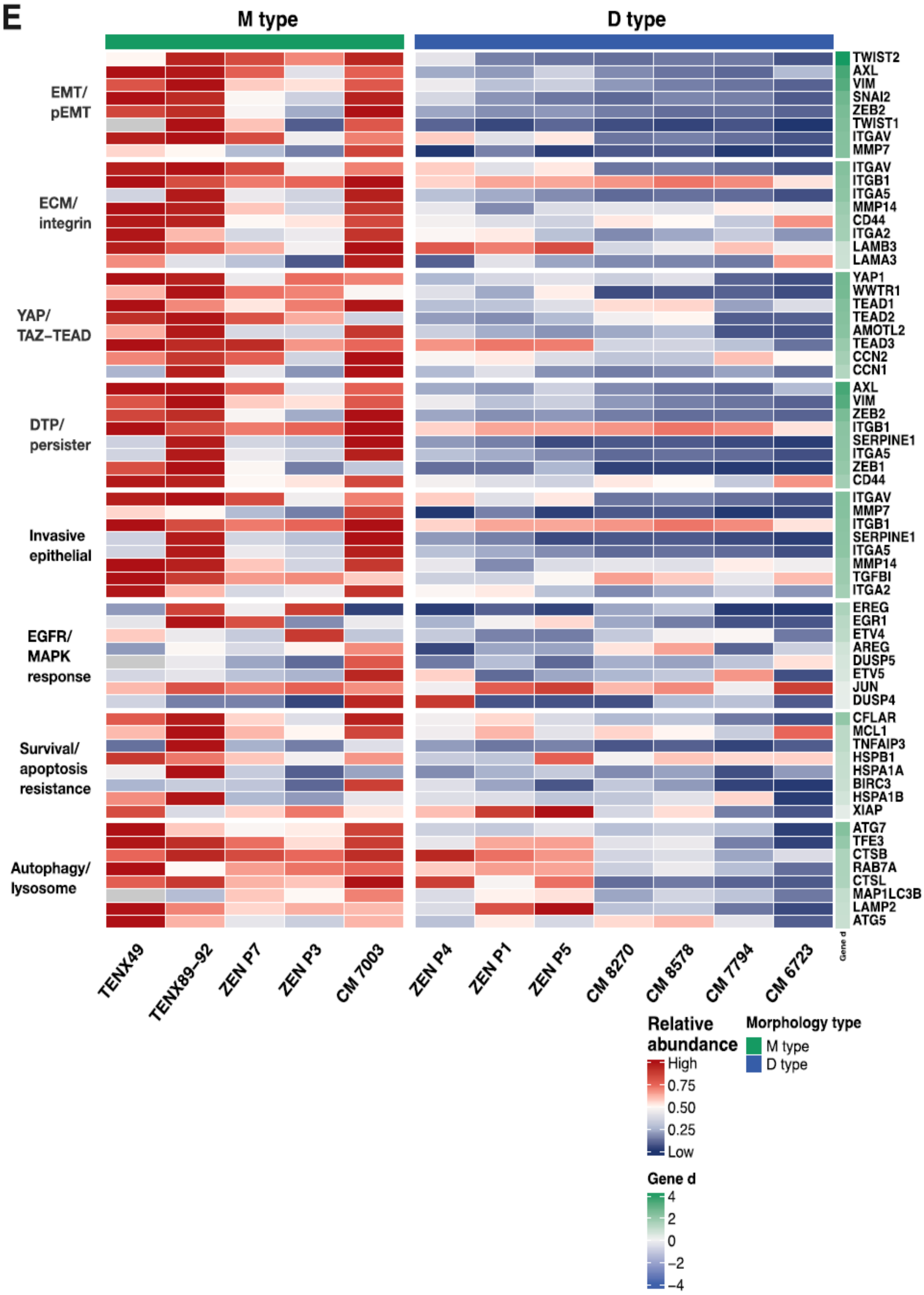
E
M type
D type
EMT/ pEMT
ECM/ integrin
YAP/ TAZ–TEAD
DTP/ persister
Invasive epithelial
EGFR/ MAPK response
Survival/ apoptosis resistance
Autophagy/ lysosome
TWIST2
AXL
VIM
SNAI2
ZEB2
TWIST1
ITGAV
MMP7
ITGAV
ITGB1
ITGA5
MMP14
CD44
ITGA2
LAMB3
LAMA3
YAP1
WWTR1
TEAD1
TEAD2
AMOTL2
TEAD3
CCN2
CCN1
AXL
VIM
ZEB2
ITGB1
SERPINE1
ITGA5
ZEB1
CD44
ITGAV
MMP7
ITGB1
SERPINE1
ITGA5
MMP14
TGFBI
ITGA2
EREG
EGR1
ETV4
AREG
DUSP5
ETV5
JUN
DUSP4
CFLAR
MCL1
TNFAIP3
HSPB1
HSPA1A
BIRC3
HSPA1B
XIAP
ATG7
TFE3
CTSB
RAB7A
CTSL
MAP1LC3B
LAMP2
ATG5
Gene d
TENX49
TENX89–92
ZEN P7
ZEN P3
CM 7003
ZEN P4
ZEN P1
ZEN P5
CM 8270
CM 8578
CM 7794
CM 6723
Relative abundance
High
0.75
0.50
0.25
Low
Morphology type
M type
D type
Gene d
4
2
0
−2
−4

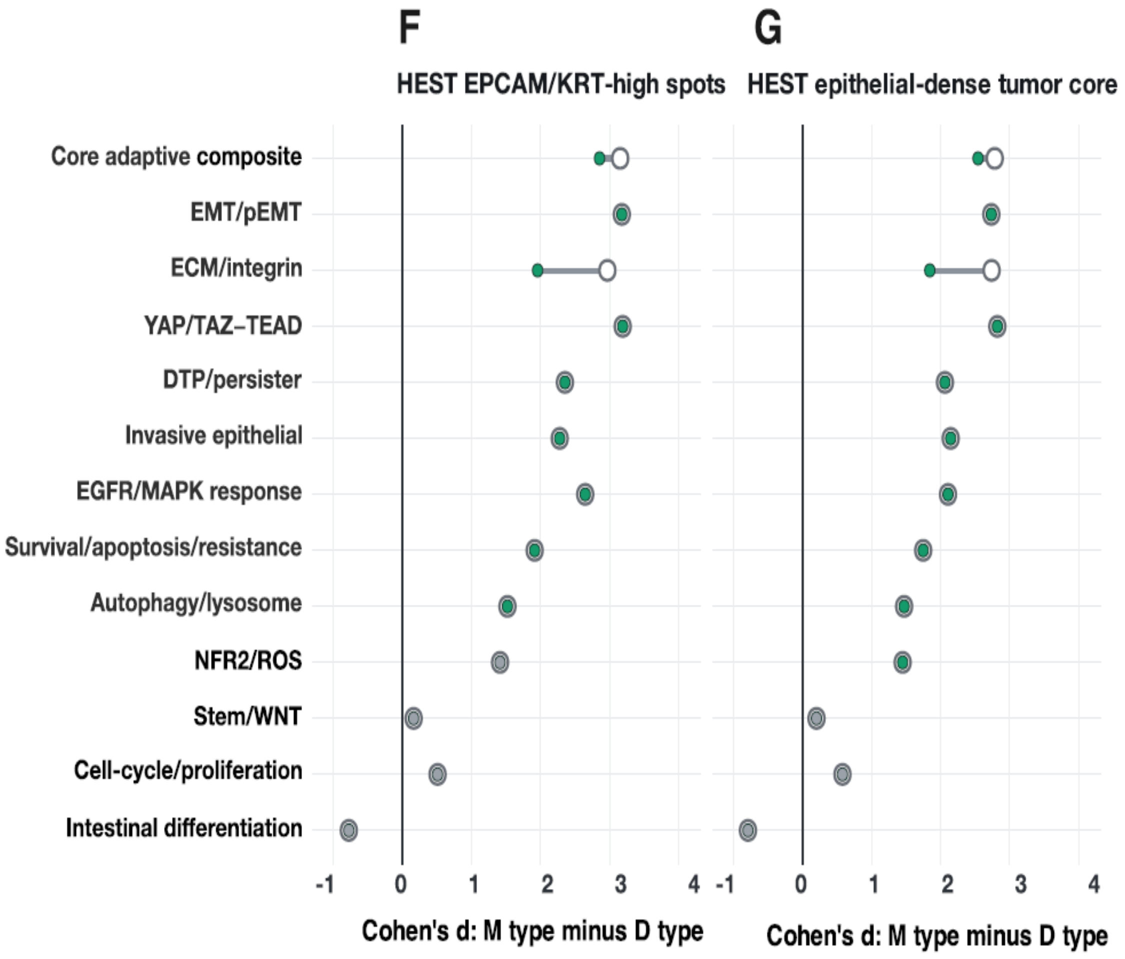

Supplementary Figure S7. Sensitivity of the HEST cancer-cell program analysis to exclusion of canonical stromal/CAF-associated genes. A. Spatial projection of the filtered cancer-cell program scores in representative M-type (TENX92) and D-type (MISC56) sections. Scores were displayed only in EPCAM/KRT-high spots; red indicates higher and blue indicates lower module relative abundance. B. Unit-level filtered module scores in five M-type and seven D-type independent patient/tissue units. Green and blue headers denote M-type and D-type, respectively; red indicates higher and blue indicates lower module scores. C. Cohen's d values for M-type versus D-type comparisons in all EPCAM/KRT-high spots and in the epithelial-dense tumor-core subset. Bar colors denote program families: red, adaptive/invasive; purple, signaling/stress; gray, stress/survival; and green, lineage/proliferation. D. Fractions of EPCAM/KRT-high spots above thresholds defined from the D-type reference distribution; green denotes M-type and blue denotes D-type. E. Constituent-gene heatmap for modules that remained higher in M-type units after gene exclusion. Red indicates higher and blue indicates lower relative abundance; green and blue headers denote M-type and D-type, respectively. F,G. Direct

comparison of the original fixed-module effects and the stromal/CAF-gene-excluded effects in all EPCAM/KRT-high spots (F) and epithelial-dense tumor-core spots (G). The same sections, spot definitions, normalization, thresholds, and unit-level statistical procedures were used in the original and filtered analyses. Open circles denote the original effects; inner circles denote the filtered effects. Green inner circles indicate a Benjamini-Hochberg-adjusted q value below 0.05 after gene exclusion. The 31 excluded genes were FN1, COL1A1, COL1A2, COL3A1, COL4A1, COL4A2, COL5A1, COL5A2, COL6A1, COL6A2, COL6A3, COL10A1, COL11A1, DCN, LUM, BGN, PDGFRA, PDGFRB, ACTA2, TAGLN, MYL9, FAP, POSTN, SPARC, TNC, VCAN, AEBP1, THY1, THBS1, THBS2, and MMP2.

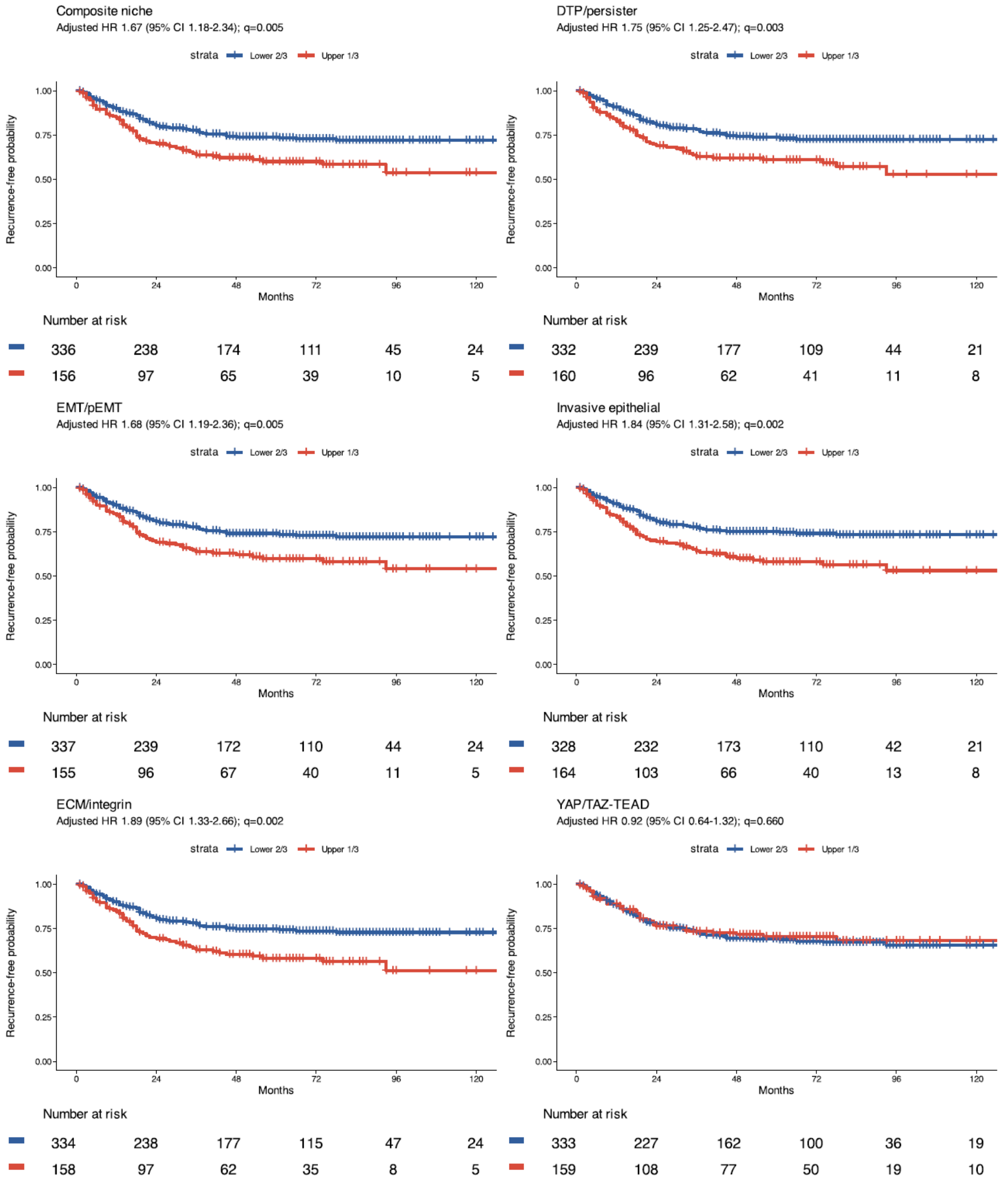

Supplementary Figure S8. Adjusted recurrence-free survival according to prespecified module-score strata in GSE39582. Kaplan-Meier curves compare the prespecified upper tertile (red) with the lower two tertiles (blue) for the composite niche, DTP/persister, EMT/pEMT, invasive epithelial, ECM/integrin, and YAP/TAZ-TEAD scores. Adjusted hazard ratios and 95% confidence intervals were obtained from Cox models including stage, tumor location, mismatch-repair status, and adjuvant chemotherapy. The displayed cutoffs were prespecified; no outcome-optimized cutoff was used. Censoring marks are shown on the curves, and numbers at risk are shown below

each panel.

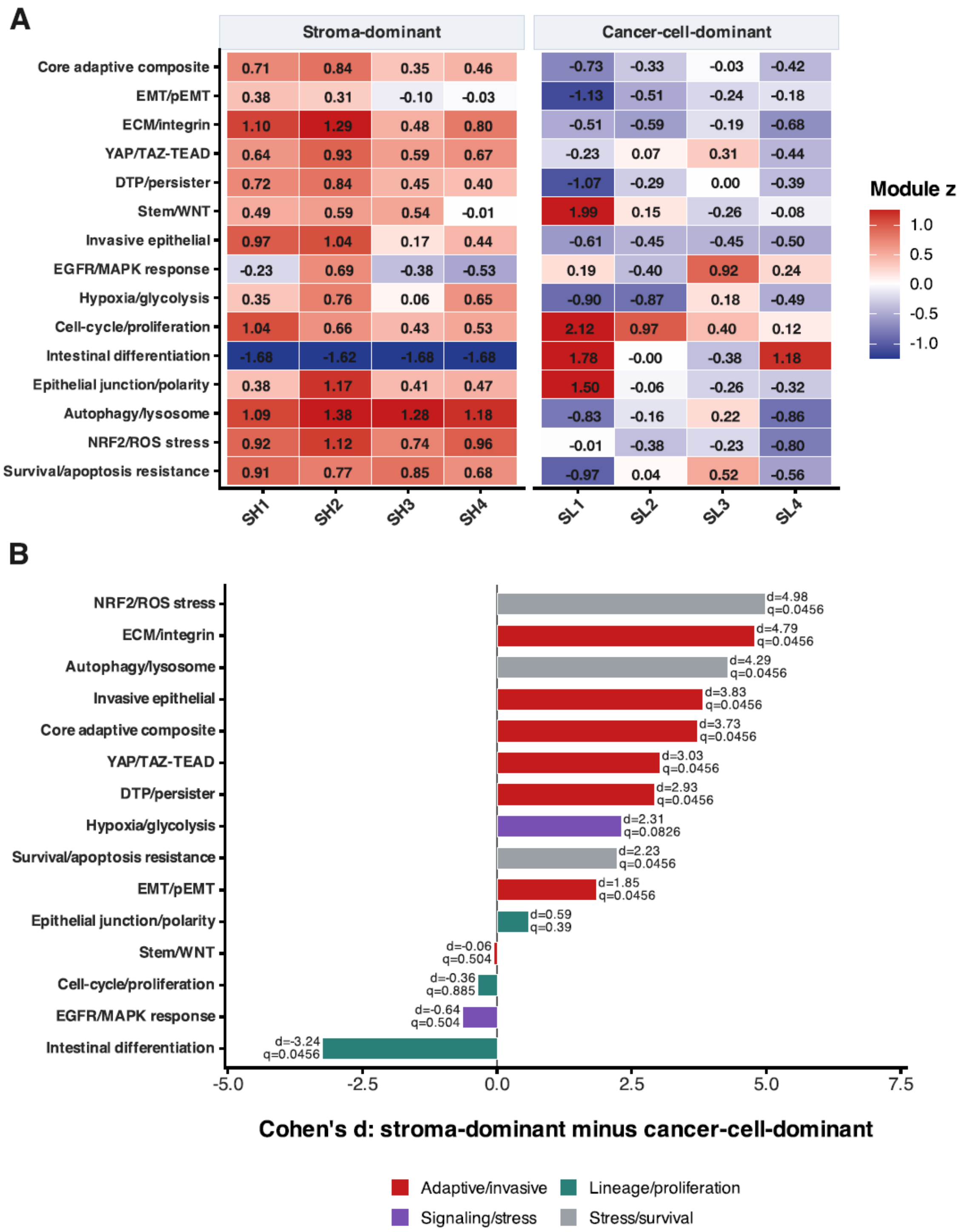
A
Stroma-dominant
Cancer-cell-dominant
Core adaptive composite
EMT/pEMT
ECM/integrin
YAP/TAZ-TEAD
DTP/persister
Stem/WNT
Invasive epithelial
EGFR/MAPK response
Hypoxia/glycolysis
Cell-cycle/proliferation
Intestinal differentiation
Epithelial junction/polarity
Autophagy/lysosome
NRF2/ROS stress
Survival/apoptosis resistance
SH1
SH2
SH3
SH4
SL1
SL2
SL3
SL4
Module z
B
Cohen's d: stroma-dominant minus cancer-cell-dominant
Adaptive/invasive
Lineage/proliferation
Signaling/stress
Stress/survival

C

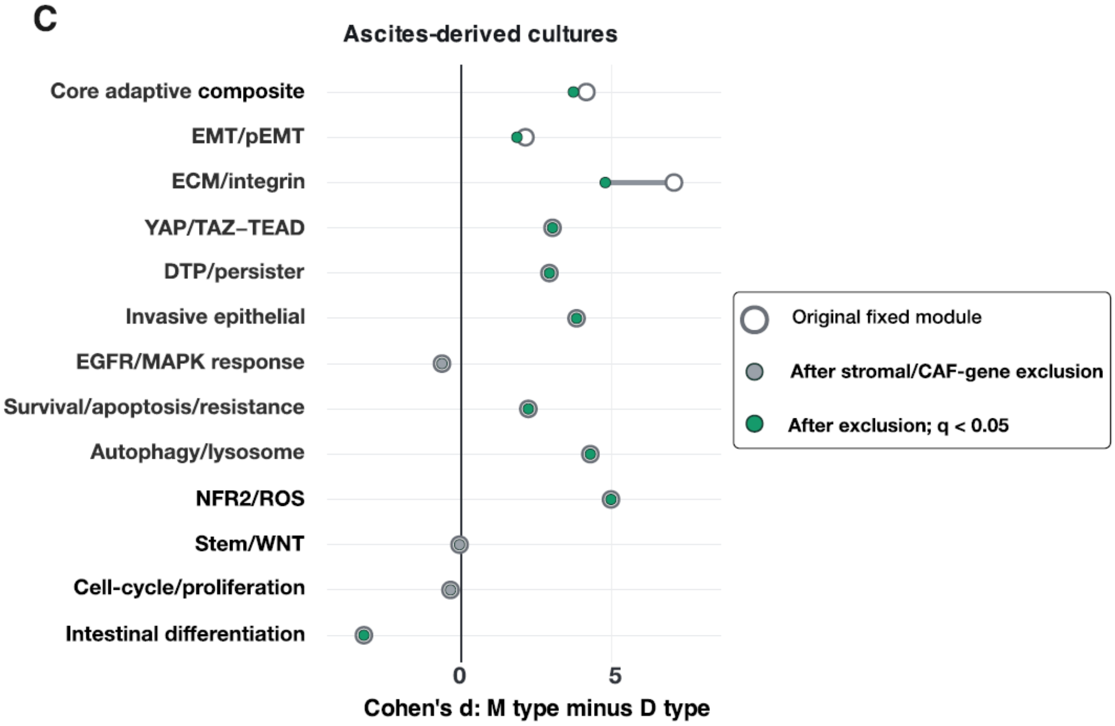

Supplementary Figure S9. Sensitivity of the ascites-culture program analysis to exclusion of canonical stromal/CAF-associated genes. A. Module-score heatmap from four stroma-dominant and four differentiated-cancer-cell-dominant ascites-derived cultures after exclusion of canonical stromal/CAF-associated genes. Red indicates higher and blue indicates lower module z-scores. B. Cohen's d values for the filtered stroma-dominant versus differentiated-cancer-cell-dominant comparison. Bar colors denote program families: red, adaptive/invasive; purple, signaling/stress; gray, stress/survival; and green, lineage/proliferation. C. Direct comparison of the original fixed-module effects and filtered effects. The samples, normalization, and statistical procedures were unchanged. Open circles denote the original effects; inner circles denote the filtered effects. Green inner circles indicate a Benjamini-Hochberg-adjusted q value below 0.05 after gene exclusion.
