# Supplementary material for: Stroma-Dominant Colorectal Cancer Harbors CAF-Rich Spatial Architecture and EMT-Associated Cancer Cell Plasticity Detectable After Dissemination
Source: Biomolecules. 2026 Aug 31;16(9):1261. doi: 10.3390/biom16091261 (PMC13604046; doi:10.3390/biom16091261)
Supplement: Supplementary file 1 [file biomolecules-16-01261-s001.zip › biomolecules-4482148-SM/Kitagawa_Biomolecules_SupplementaryMethods_R2.pdf]

Supplementary Methods

HEST-1K data source and H&E-guided section selection

Public colorectal cancer spatial transcriptomic data were obtained from the HEST-1K collection distributed as MahmoodLab/hest on Hugging Face (https://huggingface.co/datasets/MahmoodLab/hest; version 1.3.0; accessed 27 June 2026). HEST-1K was the only spatial transcriptomic resource used in this study. Whole-section H&E images were classified before transcriptomic scoring. M-type denoted stroma-dominant, mesenchymal/desmoplastic morphology, whereas D-type denoted differentiated, gland-forming, cancer-cell-dominant morphology. Initial assignments were independently reviewed by a board-certified pathologist (T.S.), and disagreements were resolved by consensus. Reviewers did not use expression data, module scores, survival outcomes, CMS labels, or ascites-culture RNA-seq results during morphology assignment. Of 22 CRC sections reviewed, two with ambiguous morphology were excluded before module projection. Formal inter-observer kappa could not be calculated retrospectively because separate reader-level calls were not retained.

The final analysis included 20 sections from 12 independent patient/tissue units. The M-type group comprised 10 sections from five units: TENX49; TENX89-92; ZEN patient 7 (ZEN36 and ZEN40); ZEN patient 3 (ZEN45); and COLON MAP 7003 (MISC66 and MISC67). The D-type group comprised 10 sections from seven units: ZEN patient 4 (ZEN39 and ZEN43); ZEN patient 1 (ZEN48 and ZEN49); ZEN patient 5 (ZEN38); COLON MAP 8270 (MISC46 and MISC48); COLON MAP 8578 (MISC43); COLON MAP 7794 (MISC56); and COLON MAP 6723 (MISC70). COLON MAP 7003 contained mixed spatial morphologies; only MISC66 and MISC67, which showed a clear M-type H&E pattern, were retained. The complete section-to-unit mapping is provided in

Supplementary Table S1.

Analyses were performed across the entire tissue-covered section; no manual transcriptomic tumor region of interest was selected. Serial or linked sections from the same specimen were treated as subsamples. Section-level summaries were averaged within each patient/tissue unit, and all primary M-type versus D-type spatial effect sizes used the unit as the independent observation (five M-type versus seven D-type units).

Section-wise compartment definition

Epithelial-content and CAF/stroma-content scores were calculated for every tissue-covered spot using predefined marker sets. The epithelial-content set comprised EPCAM, KRT8, KRT18, KRT19, KRT20, KRT7, CDH1, and CEACAM5. The CAF/stroma anchor set used for the final neighborhood analysis comprised PDGFRA, PDGFRB, DCN, LUM, BGN, COL6A1, COL6A2, SPARC, THY1, and VIM. A broader CAF/stroma marker panel (ACTA2, BGN, COL1A1, COL1A2, COL3A1, COL6A1, COL6A2, COL6A3, DCN, FAP, FBLN1, FBLN2, FN1, LUM, PDGFRB, POSTN, SPARC, TNC, and VCAN) was used for marker-level visualization and quality control. EPCAM/KRT-high spots were defined within each section as spots at or above the 60th percentile of the epithelial-content score. CAF/stroma-high spots were defined within each section as spots at or above the 80th percentile of the CAF/stroma-content score. These labels indicate compartment enrichment rather than cellular purity because one spatial spot can contain transcripts from multiple cell types.

No global batch correction or cross-dataset integration was applied to the spatial count matrices. Normalization, score standardization, thresholds, and neighborhood comparisons were calculated within section. The analysis therefore tested relative spatial

structure within each histological section and did not treat absolute expression differences among sections as global differential expression.

Prespecified module definitions and gene-set provenance

The four primary modules represented EMT/partial EMT (EMT/pEMT), ECM/integrin, YAP/TAZ-TEAD mechanotransduction, and drug-tolerant persister (DTP/persister) biology. Their biological framework and constituent genes were specified before projection into HEST-1K and were not selected from the HEST M-type versus D-type contrast or from outcome data. Genes were not reweighted according to their HEST effect size, direction of change, p value, heatmap appearance, or survival association. Dataset-specific gene availability and the prespecified HEST exclusion described below were recorded rather than replaced by surrogate genes.

The general EMT/pEMT module contained 19 genes and combined the MSigDB Hallmark epithelial-mesenchymal transition program with literature-supported partial-EMT and invasive epithelial markers: VIM, FN1, SNAI1, SNAI2, ZEB1, ZEB2, TWIST1, TWIST2, AXL, CD44, ITGA5, ITGAV, ITGB1, LAMC2, LAMB3, MMP7, MMP14, SERPINE1, and TGFBI. For HEST epithelial-side scoring and the spatial constituent-gene display, FN1 was excluded because it was also a defining CAF/stroma marker; the HEST EMT/pEMT score therefore used the remaining 18 genes. This prespecified exclusion reduced direct overlap between the epithelial-side score and the stromal compartment definition.

The ECM/integrin module contained 20 genes and was informed by Reactome integrin cell-surface interactions, KEGG ECM-receptor interaction and focal adhesion, and Gene Ontology integrin-mediated adhesion sets: COL1A1, COL1A2, COL3A1, COL6A1,

COL6A2, FN1, TNC, POSTN, SPP1, LAMA3, LAMB3, LAMC2, ITGA2, ITGA3, ITGA5, ITGAV, ITGB1, CD44, MMP2, and MMP14.

The YAP/TAZ-TEAD module contained 14 literature-supported mechanotransduction regulators and targets, including those described by Dupont et al. and Calvo et al.: YAP1, WWTR1, TEAD1, TEAD2, TEAD3, TEAD4, CCN1, CCN2, ANKRD1, AMOTL2, AJUBA, AREG, BIRC5, and MYC. CCN2 is the current gene symbol for the gene historically reported as CTGF.

The DTP/persister module contained 17 genes informed by published drug-tolerant-persister and diapause-like cancer-cell states, including the frameworks described by Sharma et al., Hangauer et al., and Rehman et al.: AXL, NGFR, KRT17, CD44, ITGA5, ITGB1, DUSP1, DUSP6, JUN, FOS, SERPINE1, VIM, ZEB1, ZEB2, HES1, NR2F1, and SQSTM1.

Gene overlap among modules was retained because the modules describe related biological programs rather than mutually exclusive classifiers. The core adaptive composite was an operational summary calculated as the unweighted mean of the four primary module scores; it introduced no additional genes and was not treated as an independently established pathway. For each dataset, only genes present in the corresponding expression matrix were used, and missing genes were not substituted.

Prespecified comparison modules included Stem/WNT, invasive epithelial, EGFR/MAPK response, hypoxia/glycolysis, cell-cycle/proliferation, intestinal differentiation, epithelial junction/polarity, autophagy/lysosome, NRF2/ROS stress, and survival/apoptosis resistance. Complete membership, gene counts, external provenance, scoring notes, and cross-dataset gene coverage for all primary and comparison modules are provided in Supplementary Tables S1-S10.

Module scoring and unit-level summaries

For whole-section EPCAM/KRT-high comparisons and tumor-core analyses, spot-level counts were library-size normalized and log transformed. Available constituent genes were averaged for each spot, and the resulting module score was standardized within section. The core adaptive composite was then calculated as the arithmetic mean of the standardized EMT/pEMT, ECM/integrin, YAP/TAZ-TEAD, and DTP/persister scores. Section-level means were calculated within the specified spot compartment and then averaged within patient/tissue unit before M-type versus D-type comparisons. Constituent-gene heatmaps displayed the prespecified genes available in the analysis rather than genes selected according to the observed group difference.

For relative-abundance heatmaps and the CAF-neighbor count gradients, each gene's log-normalized spot value was divided by its 95th-percentile reference derived from section-level mean expression across the 55-section CRC HEST panel and truncated to the interval from 0 to 1. Module relative abundance was the mean of the available scaled constituent genes. This bounded score was used for within-section spatial visualization and gradient summaries; it was not used to create a cross-platform integrated expression space.

CAF-neighborhood and spatial-gradient analysis

Two complementary neighborhood summaries were used. First, for each EPCAM/KRT-high spot, a continuous CAF-neighbor score was calculated as the mean CAF/stroma-content score of the 12 nearest spatial spots within the same section. EPCAM/KRT-high spots were divided into section-wise quintiles, and module activity was compared between the highest and lowest quintiles. Mixed-effects models tested the association

between CAF-neighbor score and cancer-cell module activity, with section modeled as a random effect. Second, immediate hexagonal neighbors were classified as CAF/stroma-high or not, and EPCAM/KRT-high spots were grouped by 0, 1, 2, or 3 or more CAF/stroma-high neighbors for the displayed count gradients. Section-level summaries were averaged within patient/tissue unit before between-morphology comparisons. Gradient source data, slopes, and across-unit summaries are provided in Supplementary Table S3.

In the complementary proximity analysis, Euclidean distance from each EPCAM/KRT-high spot to the nearest CAF/stroma-high spot was calculated within the same section. The closest and farthest section-wise quintiles were defined as CAF-proximal and CAF-distant cancer-cell-enriched regions, respectively. Because epithelial and stromal transcripts can coexist within a spot, neighborhood and proximity signals were interpreted as spatial associations at the tumor-stroma interface, not as proof of purely malignant-cell expression, signaling direction, or causality.

CAF/stromal programs and expression-inferred cancer-to-stroma axes

CAF/stromal constituent-gene abundance and candidate epithelial ligand programs were summarized at the same independent-unit level. Candidate cancer-to-stroma ligand genes were organized into prespecified biological axes. In HEST, direction labels denote paired ligand and receptor gene-set axes measured in the relevant EPCAM/KRT-high and CAF/stroma compartments; they do not establish the cellular source of a transcript, receptor activation, direct cell-cell communication, or causality. The selection rationale, complete ligand and receptor axis membership, constituent-gene values, and gene-level effects are provided in the revised Supplementary Table workbook and Supplementary

Table S4.

Tumor-core sensitivity and specificity controls

To test whether the cancer-cell-side signal could be explained entirely by adjacent stromal transcripts, the analysis was repeated in epithelial-dense tumor-core spots, defined as EPCAM/KRT-high spots with at least three immediate EPCAM/KRT-high neighbors. Primary-module effects were compared with the corresponding analysis of all EPCAM/KRT-high spots. Prespecified comparison programs, including cell-cycle/proliferation, hypoxia/glycolysis, Stem/WNT, epithelial junction/polarity, and intestinal differentiation, were evaluated to assess specificity. Source data are provided in Supplementary Table S5. Persistence of the primary-module contrast after tumor-core restriction argues against a stromal-spillover-only explanation but does not eliminate residual admixture or section-level confounding.

Stromal/CAF-gene-exclusion sensitivity analyses

To determine whether the M-type versus D-type module contrasts depended on canonical stromal genes embedded in the fixed gene sets, the HEST analyses were repeated after removing 31 stromal/CAF-associated genes: FN1, COL1A1, COL1A2, COL3A1, COL4A1, COL4A2, COL5A1, COL5A2, COL6A1, COL6A2, COL6A3, COL10A1, COL11A1, DCN, LUM, BGN, PDGFRA, PDGFRB, ACTA2, TAGLN, MYL9, FAP, POSTN, SPARC, TNC, VCAN, AEBP1, THY1, THBS1, THBS2, and MMP2. No genes were added or reweighted after exclusion. The EPCAM/KRT-high and epithelial-dense tumor-core definitions, within-section normalization, patient/tissue-unit aggregation, and statistical procedures were kept unchanged. Original and filtered spatial effects are

compared in Supplementary Figure S7.

The 31-gene stromal/CAF-exclusion panel was constructed as a conservative sensitivity filter by literature-informed curation of genes contained in the prespecified modules. Genes were included in the exclusion panel when they represented established fibroblast/CAF identity markers or extracellular-matrix structural and remodeling genes commonly attributed to fibroblast/stromal compartments in CRC. The panel was defined before the sensitivity reanalysis and independently of the M-type versus D-type comparisons, module effect sizes, survival associations, and statistical significance in the cohorts analyzed here. It was not derived from FANTOM5 or PanglaoDB and was not intended as an exhaustive CAF signature or cell-type classifier. The complete list of the 31 excluded genes is provided in Supplementary Figure S9.

Focused real-time PCR validation

Focused real-time PCR used one M-type-derived and one D-type-derived ascites-cell sample that were independent of the eight cultures included in the RNA-seq comparison. KRAS, MAP2K1, MAPK1, SMAD2, SMAD3, and TGFBR1 were measured as representative MAPK/TGF-beta pathway genes, with ACTB as the reference gene. Target expression was normalized to ACTB within each experimental run and expressed relative to the M-type-derived sample. Technical duplicate wells were summarized for each target; error bars represent technical variation. The assay was used as focused, independent support and was not treated as cohort-wide validation. The instrument-exported Ct/quantity measurements and normalized summaries are provided in the Supplementary Table workbook.

Immunohistochemical quantification

CD44v6 and VIM immunohistochemistry was quantified using a custom browser-based image-analysis workflow with hematoxylin-DAB color deconvolution. CD44v6 was measured within manually selected tumor epithelial regions of interest (ROIs) from primary tumors and matched metastatic lymph-node lesions. VIM was measured within manually selected stromal ROIs from representative M-type and D-type primary tumors. Necrosis, mucin pools, tissue folds, blank areas, and non-target tissue were excluded. Fixed DAB cutoffs were applied across images stained with the same antibody. For each ROI, DAB-positive area and an intensity-weighted H-like score were calculated. The H-like score was defined as 1 x weak-positive area (%) + 2 x moderate-positive area (%) + 3 x strong-positive area (%). Multiple ROIs were averaged to obtain one case-level value. CD44v6 comparisons used case-level means from three M-type and three D-type cases; Cohen's d and an exact two-sided permutation p value were reported, with descriptive interpretation because of the small number of cases. VIM was summarized descriptively from three stromal ROIs in one representative case per morphology group.

Statistical analysis and interpretation

The patient/tissue unit was the independent observation for spatial M-type versus D-type comparisons ($n = 5$ and $n = 7$, respectively). Cohen's d was calculated as the difference between the M-type and D-type unit means divided by the pooled unit-level standard deviation; positive values indicate higher abundance or activity in M-type units. Exact permutation, Wilcoxon, and mixed-effects tests were used as specified for each analysis. Benjamini-Hochberg correction was applied separately within the prespecified spatial-module, CAF-neighborhood, ligand/receptor-axis, ascites-culture-module, and survival-

module families. The four primary module scores and their operational composite in EPCAM/KRT-high spots were the primary spatial outcomes. All tests were two-sided. Spot- and section-level observations were not counted as independent biological replicates for between-group effect sizes.

The HEST-1K results are interpreted as exploratory unit-level associations between consensus H&E morphology and spatial transcriptional programs. H&E grouping was completed independently of the calculated module outcomes, but the analysis remains limited by the small number of independent units, residual differences among sections, the mixed-cell composition of spatial spots, and the absence of a second spatial cohort analyzed with the identical selection and scoring procedure. Concordant findings in ascites-derived cultures provide orthogonal support for morphology-associated programs but do not constitute an independent spatial validation or proof of a purified malignant-cell-intrinsic state.

Multivariable survival analyses

TCGA overall survival was analyzed in complete cases using Cox proportional-hazards models. The primary morphology model compared M-pattern with D-pattern tumors and adjusted for age, stage (I/II versus III/IV), primary site (COAD versus READ), MANTIS microsatellite-instability status, and ABSOLUTE tumor purity (n = 426; 77 deaths). A sensitivity model additionally included CMS subtype (n = 275; 58 deaths). Separate joint models included H&E morphology, one continuous module score, and the same clinical covariates. Adequately complete systemic-treatment data were not available for the TCGA working cohort.

GSE39582 recurrence-free survival was analyzed with one continuous module score per

Cox model, adjusted for stage, tumor location, mismatch-repair status, and adjuvant chemotherapy (n = 492; 140 recurrence events). Original mean-constituent-gene z scores were entered directly without a second standardization. Benjamini-Hochberg correction was applied across the prespecified module models. Kaplan-Meier curves used a prespecified upper-tertile versus lower-two-thirds comparison; no outcome-optimized cutoff was used. Proportional-hazards assumptions were evaluated using scaled Schoenfeld residuals. Full effect estimates are reported in Table 1, and adjusted Kaplan-Meier analyses are shown in Supplementary Figure S8.

Supplementary source-data organization

Supplementary Tables S1-S5 retain the original spatial source-data organization: the section-to-independent-unit map; cancer-cell-enriched constituent-gene and module results; CAF-neighbor gradients; candidate cancer-to-stroma ligand and CAF/stromal results; and tumor-core robustness controls. Supplementary Tables S6-S10 provide the additional fixed-module definitions and provenance, ligand-receptor axis definitions, stromal/CAF-gene-exclusion outputs, and focused real-time PCR measurements. Table 1 reports the multivariable prognostic models, and Table 2 reports the SH1-SH4 and SL1-SL4 culture metadata. Supplementary Figure S7 shows the HEST stromal/CAF-gene-exclusion analysis, Supplementary Figure S8 shows adjusted GSE39582 recurrence analyses, and Supplementary Figure S9 shows the corresponding ascites-culture sensitivity analysis.
